# Utilizing Synergistic Potential of Mitochondria-Targeting Drugs for Leukemia Therapy

**DOI:** 10.3389/fonc.2020.00435

**Published:** 2020-04-03

**Authors:** Svetlana B. Panina, Jingqi Pei, Natalia Baran, Marina Konopleva, Natalia V. Kirienko

**Affiliations:** ^1^Department of BioSciences, Rice University, Houston, TX, United States; ^2^Department of Leukemia, The University of Texas MD Anderson Cancer Center, Houston, TX, United States

**Keywords:** mitochondria-targeted drugs, mitocans, AML, leukemia treatment, targeted drug screening, synergistic drug combinations

## Abstract

Acute myeloid leukemia (AML) is an aggressive group of cancers with high mortality rates and significant relapse risks. Current treatments are insufficient, and new therapies are needed. Recent discoveries suggest that AML may be particularly sensitive to chemotherapeutics that target mitochondria. To further investigate this sensitivity, six compounds that target mitochondria [IACS-010759, rotenone, cytarabine, etoposide, ABT-199 (venetoclax), and carbonyl cyanide *m*-chlorophenylhydrazone] were each paired with six compounds with other activities, including tyrosine kinase inhibitors (midostaurin and dasatinib), glycolytic inhibitors (2-deoxy-D-glucose, 3-bromopyruvate, and lonidamine), and the microtubule destabilizer vinorelbine. The 36 resulting drug combinations were tested for synergistic cytotoxicity against MOLM-13 and OCI-AML2 AML cell lines. Four combinations (IACS-010759 with vinorelbine, rotenone with 2-deoxy-D-glucose, carbonyl cyanide *m*-chlorophenylhydrazone with dasatinib, and venetoclax with lonidamine) showed synergistic cytotoxicity in both AML cell lines and were selective for tumor cells, as survival of healthy PBMCs was dramatically higher. Among these drug pairs, IACS-010759/vinorelbine decreased ATP level and impaired mitochondrial respiration and coupling efficiency most profoundly. Some of these four treatments were also effective in K-562, KU812 (chronic myelogenous leukemia) and CCRF-CEM, MOLT-4 (acute lymphoblastic leukemia) cells, suggesting that these treatments may have value in treating other forms of leukemia. Finally, two of the four combinations retained high synergy and strong selectivity in primary AML cells from patient samples, supporting the potential of these treatments for patients.

## Introduction

Acute myeloid leukemia (AML) is a heterogeneous group of aggressive hematological malignancies that are characterized by the proliferation of undifferentiated or partially differentiated myelogenous blast cells. Despite efforts to improve treatment, front-line drug regimens for AML have remained essentially unchanged for 20 years, with the exception of very recent approvals of targeted therapies for a few patient populations (e.g., those with IDH1 or FLT3 mutations). Current front-line treatment for AML, called induction and consolidation, utilizes a combination of doxorubicin, daunorubicin, or idarubicin with cytarabine (1). Although this treatment has some success in young adults, elderly patients continue to exhibit poor treatment outcomes due to drug toxicity and/or comorbidities, with fewer than 10% of patients surviving more than 2 years (2). In addition, relapse is a significant problem in AML, particularly due to a recurring population of leukemic cells that often remain resistant to frontline treatment or have acquired drug resistance (3). New, more effective treatments are clearly necessary.

One possible option is the development of new combinatorial therapeutic regimens. Combinatorial drug therapy may resolve some limitations of single drugs. For example, synergistic drug pairs may amplify each other's activity, leading to more substantial effects against leukemic cells while drug concentrations are lower than conventional monotherapies. In turn, these lower concentrations may reduce drug toxicity in healthy bystander cells (4). Combination therapies are usually designed as pairs of drugs from different pools of available treatments and that target non-overlapping biological pathways (preferably those that already have regulatory approval). This approach has already been shown to be fruitful for identifying combinations that are both effective (show strong cytotoxicity in cancer cells) and selective (show little or no killing of normal cells) in hematologic malignancies (5). However, the search for synergistic combinations is a major challenge since the number of possible combinations scales exponentially, making screening expensive and empirical validation laborious (6, 7). Due to these difficulties, it is critical that careful consideration be used for selecting the most promising targets for initial testing.

Historically, the observations of Otto Warburg (i.e., that cancers shift from oxidative phosphorylation (OxPhos) to glycolysis to meet their energy needs) have led cancer researchers to largely disregard the importance of mitochondrial metabolism in cancer. Were it not for their functions in regulating programmed cell death pathways, mitochondria may have been completely overlooked by cancer researchers. There has been a recent renaissance in the study of OxPhos and its role in providing the energy and building blocks required for cell division in cancers (8–11). For example, contrary to Warburg's model, some cancers show increased mitochondrial dependence on glucose and glutamine (12, 13). Depriving the tumor cells of these substances causes rapid cancer cell death, suggesting that this may have potential value as a cancer treatment strategy. This has been cited as a justification for the development mitochondria-targeting compounds (14, 15). Drugs targeting many other aspects of mitochondrial biology, such as the generation of reactive oxygen species, the electron transport chain, or the mitochondrial permeability transition pore have also been shown to kill tumor cells (16–22). “Mitocan” has been proposed as a generalized term for compounds that target mitochondria to disrupt tumor cells (23). Using both bioinformatics and cell culture studies, we recently determined that AML cells are particularly sensitive to mitocans, whether alone or in combination, and exposure to these compounds can effectively kill these cells (24). Interestingly, the drugs used for induction and consolidation (cytarabine and daunorubicin or doxorubicin) both have underappreciated effects on mitochondria (25, 26). This is consistent with our previous findings.

In this study, we used fluorescent cell labeling and automated counting to quickly and accurately determine the cytotoxicity of 36 drug combinations, each comprised of one mitocan and a drug from one of three other classes (tyrosine kinase inhibitors, a microtubule inhibitor, or anti-glycolytic drugs). To determine potential drug synergy, each of the 36 combinations was tested against two different AML cell lines (OCI-AML2 and MOLM-13) at several different dose combinations. Four drug combinations showed significant synergistic cytotoxicity and strong selectivity (i.e., >50% difference in cytotoxicity when compared to healthy PBMCs). Two of these combinations (IACS-010759/vinorelbine and rotenone/2-deoxyglucose) demonstrated strong efficacy and good selectivity against primary cells derived from AML patients. Mechanistically, these two drug pairs, especially IACS-010759/vinorelbine, were shown to efficiently inhibit mitochondrial respiration and rapidly deplete ATP levels. These combinations were also synergistic and selective against leukemia cell lines from patients with chronic myelogenous leukemia (CML) or acute lymphoblastic leukemia (ALL). These findings support the potential of these combinations for future therapeutic development and optimization and reinforce the value of this approach.

## Materials and Methods

### Cell Cultures and Primary AML Samples

AML (MOLM-13, OCI-AML2), CML (K-562, KU812), and ALL (CCRF-CEM, MOLT-4) cell lines were purchased from ATCC (Manassas, VA, USA). OCI-AML2 and MOLM-13 cell lines were used as representative AML cell lines for primary screening based on their difference in genetic background, specifically FTL3-ITD status. A complete list of cell lines studied is available as Table S1.

Peripheral blood samples from patients with AML (*n* = 16) were collected during standard diagnostic procedures after informed consent was obtained in accordance with the Institutional Review Board (IRB) regulations (IRB protocol PA13-1025 for UTMDACC and IRB-FY2019-143 for Rice University). The study design adhered to the tenets of the Declaration of Helsinki and was approved by the ethics committees of the participating institutions before its initiation. Basic characteristics of the patients from which samples were derived are shown in Table S2. Patients' samples AML1-12 were used for cytotoxicity assays, and samples AML13-18 were used for bioenergetic measurements.

Peripheral blood mononuclear cells (PBMCs) from blood donations from healthy blood donors were used as healthy counterpart for AML cells. Healthy PBMCs and primary AML cells were isolated using Leukosep tubes (Sigma-Aldrich, St. Louis, MO, USA) and Ficoll-Paque™ (Sigma-Aldrich) following the manufacturer's instructions. For all experiments, healthy PBMCs were used either shortly after isolation or rested overnight after thawing. Primary AML samples were used immediately after isolation.

All leukemia cell lines were routinely cultured in RPMI-1640 media, supplemented with 2 mM L-glutamine (Sigma-Aldrich) and 10% HyClone fetal bovine serum, FBS (GE Healthcare, Pittsburgh, PA, USA) at 37°C in a humidified 5% CO_2_ atmosphere. Primary AML samples and healthy PBMCs were maintained in RPMI-1640 media with 10% FBS for 3–4 days. Penicillin and streptomycin mix (Gibco, Gaithersburg, MD, USA), were added to the media at a final concentration of 1%.

### Treatments and Cytotoxicity Assays

**Figure 1:**
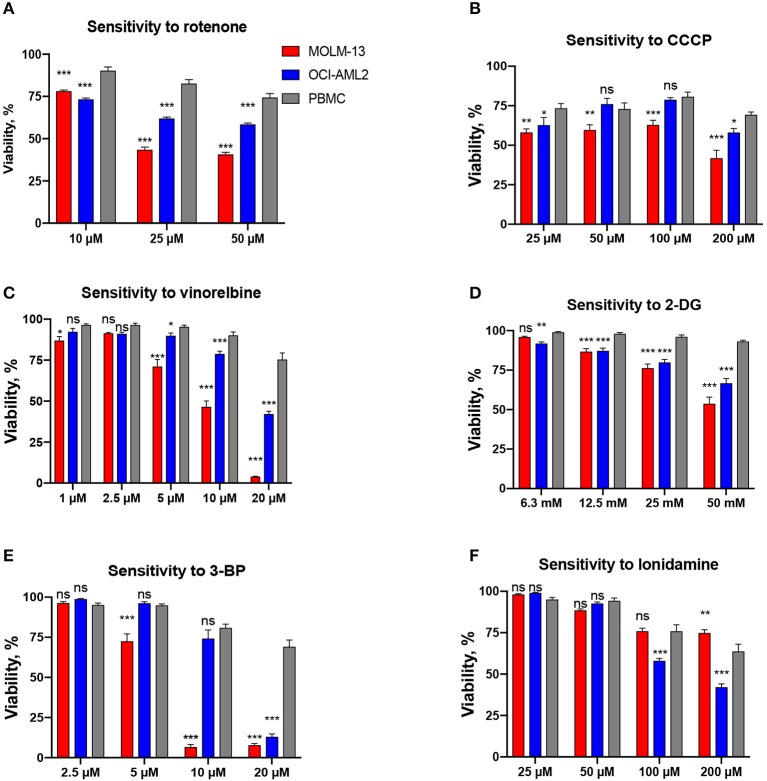
Drugs included in the screen based on their selectivity toward AML cells. Survival of AML cells (OCI-AML2 or MOLM-13) or healthy PBMCs following 24 h treatment with **(A)** rotenone, **(B)** CCCP, **(C)** vinorelbine, **(D)** 2-deoxy-D-glucose, **(E)** 3-bromopyruvate, **(F)** lonidamine. The average of at least three independent replicates ± SEM is shown. Significance of difference in survival (AML cells vs. PBMCs) was assessed via Student's *t*-test. ^***^p < 0.001; ^**^p < 0.01; ^*^p < 0.05; ns: *p* > 0.05.

Combinations based on mitocans with different mechanism of action (OxPhos inhibitors, DNA-targeted and pro-apoptotic drugs, uncouplers) and other classes of chemotherapies (tyrosine kinase inhibitors (TKI)/anti-microtubule/anti-glycolytic agents) were tested. The drugs were chosen based on either their known efficacy against AML (Table S3) or their selective cytotoxicity against AML cells compared to healthy PBMCs at several doses tested (Figure 1). This selectivity has been established by preliminary cytotoxicity assays.

The stock solutions of rotenone/RT (Ark Pharm Inc., Arlington Heights, IL, USA), IACS-010759/IACS (ThermoFisher, Waltham, MA, USA), cytarabine/ara-C (Accela, San Diego, CA, USA), etoposide/ET (Chem-Impex, Wood Dale, IL, USA), ABT-199 (ThermoFisher), carbonyl cyanide *m*-chlorophenylhydrazone/CCCP (Sigma-Aldrich), midostaurin/MID (MedChemExpress, Monmouth Junction, NJ, USA), dasatinib/DAS (LC Laboratories, Woburn, MA, USA), vinorelbine/VIN (Biotang Inc., Lexington, MA, USA), lonidamine/LND (Tocris, Minneapolis, MN, USA), and pan-caspase inhibitor z-VAD-fmk (Apexbio Technology, Houston, TX, USA) were dissolved in DMSO, aliquoted, and stored at −20°C. The glycolytic inhibitors 2-deoxy-*D*-glucose/2-DG (Chem-Impex), 3-bromopyruvate/3-BP (Alfa Aesar, Haverhill, MA, USA), and the autophagic inhibitor 3-methyladenine/3-MA (AdipoGen Life Sciences, San Diego, CA, USA) were prepared as fresh solutions in serum-free RPMI-1640 media just prior to use.

For cytotoxicity assays, cells were seeded at 15,000/well (leukemia cell lines), 15–20,000/well (primary AML samples) or 50,000/well (healthy PBMCs) in flat bottom, black 96-well plates in serum-free RPMI-1640 media and immediately treated with one or two drugs in a total volume of 100 μl for 24 h. Treatment concentrations for cell lines and primary cells are shown in **Tables S4, S5**, respectively. Drug cytotoxicity was determined in serum-free media to prevent reaction of serum components with drugs, as this has been shown to influence cytotoxicity (27, 28). All viability rates were normalized to corresponding DMSO-control or media-control (in case of such drugs as 2-DG or 3-BP) wells. Since there were different DMSO amounts in the wells depending on final drug1/drug2 concentrations, we used highest DMSO dose as corresponding solvent-control. The DMSO concentrations in the incubation mixtures or solvent-control wells never exceeded 0.5% (v/v). Treatment duration of 24 h was chosen based on preliminary time-course experiments and published studies on combination treatment of leukemia cells (29, 30). Each experiment was carried out at least three times independently, excluding treatment of primary AML samples, which was conducted in 1–3 replicates due to their short-term maintenance in culture.

To determine cytotoxicity, we used a differential nuclear staining assay that utilizes two fluorescent DNA intercalators, Hoechst 33342 and propidium iodide (PI). This assay has been shown to be robust, straightforward, reliable, and suitable for primary and secondary screens of compounds with potential cytotoxic activity (31). In brief, after 24 h of treatment, cells were stained with 20 mM (1:1,000) Hoechst 33342 (ThermoFisher) and PI (ThermoFisher) at a final concentration of either 5 μg/ml (leukemia cells, primary AML samples) or 1 μg/ml (healthy PBMCs) for 15 min at 37°C. Propidium iodide final concentration was chosen based on target cell viability in media-control wells (above ~90% for leukemia cell lines, above ~70% for primary normal PBMCs). Then, cells were centrifuged directly in plates (1,000 rpm for 4 min) and immediately visualized using DAPI and Texas Red filter sets for a Cytation5 Cell Imaging Multi-Mode Reader (BioTek, Winooski, VT, USA). Images were taken at 4× magnification. Analysis of images was performed using Gen5 software v 3.00. The steps were as follows: image preprocessing (dark background subtraction), nuclear mask (threshold value DAPI ≥ 6,000) based on Hoechst 33342 signal, subpopulation analysis based on PI staining (threshold value Texas Red ≥ 5,000), recording of cell counts and calculation of viability. Examples of images taken are shown in Figure S1.

For determination of cytotoxicity using trypan blue exclusion, cells were treated with a single drug or a combination at a final density of 5 × 10^5^ leukemia cells/ml or 7.5 × 10^5^ PBMCs/ml in a serum-free RPMI-1640 media for 24 h. Survival rates were assessed using 0.4% trypan blue (Gibco) exclusion using Automated Cell Counter Countess™ II FL (ThermoFisher). All viability rates were normalized to the corresponding solvent-control wells.

### ATP Measurement

For ATP level determination, cells were seeded at 500,000/mL (leukemia cells) or 750,000/mL (healthy PBMCs) and treated for 16 h. ATP levels were measured using a bioluminescence assay (Molecular Probes™ ATP Determination Kit, ThermoFisher) according to manufacturer's instructions. A Cytation5 multi-mode plate reader (BioTek) was used to measure luminescence. ATP measurements were normalized to the total cell number.

A highly sensitive cellular viability assay Cell-Titer Glo^R^ (Promega, Madison, WI, USA) was used to compare changes in ATP level between different cell types after short treatment (2 h) with selected drug combinations at 1/1,000 dose of maximal selectivity.

### Bioenergetic Measurements

Real-time mitochondrial function in AML cells and healthy PBMCs was assessed using the Seahorse XF Cell Mito Stress Test kit (Agilent Technologies, Santa Clara, CA, USA) on Seahorse XFe96 Extracellular Flux Analyzer (Agilent Technologies). Briefly, cells, untreated or treated for 2 h with selected drug combinations at the 1/1,000 dose of maximal selectivity, were plated on a Cell-Tak (ThermoFisher) coated XF96 96-well microplate using XF base media, supplemented with 1 mM pyruvate, 2 mM L-glutamine, 5 mM glucose. OCR measurements were recorded after port injection starting with oligomycin (1.5 μM) followed by FCCP (1 μM), and lastly, a combination of antimycin A and rotenone (0.5 μM). All measurements were normalized to the number of viable cells. Basal respiration, ATP-coupled respiration, maximal and spare capacities, ECAR values, as well as non-mitochondrial respiration were recorded per each condition. The results were analyzed in Seahorse Mito Stress Test Generator (Agilent Technologies).

### Drug Combination Landscapes and Statistical Analyses

Statistical analyses were performed using RStudio v. 1.1.453 and GraphPad Prism v.8. To assess potential synergy of drug pairs against leukemia cells, we built 5^*^5 drug combination landscapes using Bioconductor package “synergyfinder” and its Bliss model (32), using multiple-ray design (33).

For every landscape, we treated cells with serial 2-fold dilutions of drug(s). Maximum testing concentration (MTC) for each drug was defined as either the dose resulting in 30–50% survival for at least one AML cell line (MOLM-13/OCI-AML2) or the dose corresponding to the cells' maximum tolerance for DMSO (0.5% final) or the limit of drug solubility (4×), which was established by preliminary single drug treatments (Table S4). If a single drug MTC that would result in <25% difference in survival between MOLM-13 and OCI-AML2 cells could be determined, that concentration was used as the MTC for both cell lines. An appropriate MTC was identified in this way for 9 of the 12 drugs tested (all except etoposide, vinorelbine, and 3-bromopyruvate). The same rules were used for ALL/CML cell lines (Table S4) and primary AML cells (Table S5).

Maximal synergy coefficients were extracted from each landscape and recorded. We concluded a drug combination to be strongly synergetic against AML cells when the average value of maximal synergy coefficient from all biological replicates (*n* = 3–4) was equal to or higher than 20 in at least one cell line and equal to or higher than 10 in both cell lines. The drug combinations meeting this cutoff, were tested for toxicity against healthy blood cells at these doses. For comparing AML vs. healthy PBMCs, two-tailed *t*-tests were used. *p* < 0.05 was considered as significant. From all landscape coordinates, only those conditions where PBMCs survived significantly better than both AML cell lines were chosen for further calculation of maximal difference in survival between AML cells and PBMCs. We concluded drug combinations to be highly selective against AML when the average % maximal difference in survival was higher than 50%. An example calculation can be found in Table S6.

Group comparisons were performed using Student's *t*-test or ANOVA with subsequent pairwise Fisher LSD tests. Correlations were estimated using Pearson *r* coefficient. *p* < 0.05 were considered as significant.

## Results

### Primary Screening Identifies Drug Combinations With Synergistic Cytotoxicity

In a previous study, we determined that leukemia cells were significantly more sensitive to mitochondria-targeted drugs than other cancer types (24). In addition, the combination of mitocans with the glycolytic inhibitor 2-deoxy-D-glucose exhibited synergy in killing leukemia cells (24). To explore the potential for mitocan-driven synergetic cell killing, we selected 6 mitocans targeting different mitochondrial functions (OxPhos, mitochondrial membrane potential, mtDNA replication, and apoptosis) and tested their combination with six complementary drugs (Table S3). Mitocans were selected based on their presence in current chemotherapeutic regimens for AML, such as cytarabine (1) or ABT-199 (34), promising clinical trials for patients with leukemia, such as IACS-010759 (35), etoposide (36), or preliminary and published data, indicating selectivity to AML, such as rotenone and CCCP (24). Complementary drugs included tyrosine-kinase inhibitors [midostaurin (37) and dasatinib (38), both of which are used in leukemia patients], glycolytic inhibitors (2-deoxy-D-glucose, 3-bromopyruvate, and lonidamine), and a microtubule destabilizer [vinorelbine (39)] (Table S3). These drugs were chosen based on results of preliminary cytotoxicity experiments showing their selectivity toward AML cells compared to healthy blood cells (Figure 1). While cytarabine was included in our screening efforts, the pronounced fluorescence of anthracycline compounds (doxorubicin, daunorubicin, etc.) precluded their inclusion in our assays. Each of the 12 drugs was tested alone, using several concentrations, in two AML cell lines, MOLM-13 and OCI-AML2. A nuclear staining assay using two fluorescent DNA intercalators, Hoechst 33342 and propidium iodide (PI), was chosen for evaluating cytotoxicity for this screen since it does not rely upon mitochondrial activity, unlike other common assays [e.g., MTT assay (26)]. This nuclear staining assay has been shown to be robust, straightforward, reliable, and suitable for primary and secondary screens of compounds with potential cytotoxic activity (31) (Figure S1). For each compound/cell line combination, the concentration needed to reduce cell survival to 30–50% (see Figure 1 for an example graph) was determined. This concentration will be referred to as the maximum testing concentration, or MTC. When possible (i.e., when difference in cell survival for MOLM-13 and OCI-AML2 for a given MTC value was <25%), same MTC values were used for both cell lines. However, in some cases substantial differences in sensitivity were observed (e.g., vinorelbine exhibited considerably higher toxicity against MOLM-13 than OCI-AML2 cells). In these cases, individual MTC values were chosen. To optimize synergy tests, a CCCP/2-deoxyglucose combination, previously found to be synergistic, was tested at several time points (Figure S2). 24 h was chosen for the primary screen as it showed the best effect.

**Figure 2:**
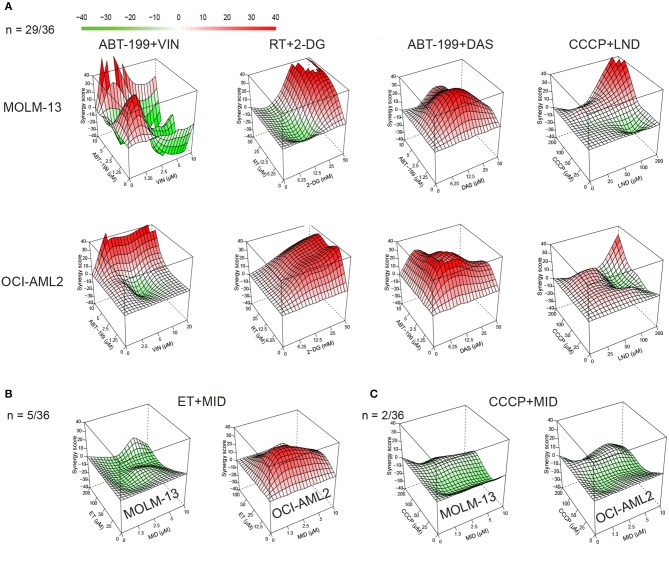
Synergy landscapes for mitocan-based AML drug combinations. Drug combination landscapes were built using Bioconductor package “synergyfinder.” One representative replicate (with maximal synergy closest to its average value) is shown. **(A)** Examples of drug combinations (*n* = 29/36), synergistic against AML cell lines. Shown are drug combinations having highest (>70) cumulative synergy against MOLM-13 (top) and OCI-AML2 (bottom) cells. **(B)** Example of drug combination with opposite profiles in MOLM-13 and OCI-AML2 (*n* = 5/36). **(C)** Examples of antagonistic drug combinations for MOLM-13 (left) and OCI-AML2 (right) cells (*n* = 2/36). Drug combination landscapes: *z-axis*, synergy score (ranges from −40, green, to +40, red); *x/y-axes*, drug1/drug2 concentration range, respectively. For specific drug names and drug concentrations, refer to Table S4.

For synergy tests, all 36 pairwise combinations were made by combining compounds such that each constituent was present at its MTC. We also made three serial 2-fold dilutions from the MTC mixture (see Table S4 for the list of concentrations used for each drug). Combinations were then tested against MOLM-13 and OCI-AML2 cell lines, along with vehicle-only controls. The resulting 5 × 5 matrices (4 dilutions + no drug control) were used to build synergy landscapes (Figure 2) using a Bliss model and the “synergyfinder” package in Bioconductor (32). Next, maximum synergy was determined for each cell line/treatment combination for at least three biological replicates, and the mean was calculated. Despite the differences in genotype, a significant correlation was observed between synergy scores in MOLM-13 and OCI-AML2 cells (*r* = 0.571, *p* < 0.01); most times (*n* = 29/36), drug combinations were synergistic in both cell lines. This bolsters the idea that these treatments may be effective on a variety of AML subtypes. It is worth noting that a few differences were observed. For example, some combinations (*n* = 5/36) showed synergy in one AML line but antagonism in the other (Figure 2B) and two combinations showed neutrality or antagonism in both cell lines (e.g., CCCP with midostaurin) (Figure 2C).

Finally, the sum of the maximum synergy scores for the two cell lines was calculated for each drug combination. Drug combinations were then ranked in decreasing order of effect (Table 1). Twenty-two of the 36 combinations tested passed our predefined cutoff criteria, as follows: First, the maximal synergy for each cell line had to be >10. Second, the maximal synergy for at least one cell line should be >20. It is worth noting that the most effective combinations (those with cumulative synergy >70 due to high synergy in both cell lines) exhibited diverse molecular targets (Table 1, Figure 2A). Toward the bottom half of the ranked list, there were several combinations that only demonstrated synergy in one of the cell lines, but not the other, such as etoposide/vinorelbine and etoposide/midostaurin (synergy in OCI-AML2), or cytarabine/3-bromopyruvate (synergy in MOLM-13) (Figure 2B, Table 1). Still further down the list were combinations antagonistic in one or both of the cell lines (Figure 2C). Interestingly, dasatinib and midostaurin are both tyrosine kinase inhibitors, but their combination with CCCP had different results: the former showed strong synergy while the latter was antagonistic, which suggests that while it is likely that the combination of anti-cancer chemotherapeutics will result in some synergy, predicting which compound combinations will exhibit synergy solely on the basis of their known mechanisms will be difficult.

**Table 1:** Mitocan-based drug combinations ranked in order of decreasing combined synergy coefficient.

**Rank**	**Drug combination**	**Sum of maximal synergy coefficients in both cell lines**	**Maximal synergy^a^, mean ± SEM, MOLM-13**	**Maximal synergy^a^, mean ± SEM, OCI-AML2**
1.	**ABT-199/VIN**	91.5	40.9 ± 9.1	50.5 ± 3.0
2.	**RT/2-DG**	77.1	40.1 ± 5.7	36.9 ± 2.1
3.	**ABT-199/DAS**	75.9	38.4 ± 0.5	37.6 ± 2.6
4.	**CCCP/LND**	74.8	47.4 ± 9.4	27.4 ± 3.3
5.	**ABT-199/3-BP**	69.5	28.0 ± 5.1	41.4 ± 5.7
6.	**IACS-010759/VIN**	68.7	37.7 ± 5.6	31.0 ± 6.1
7.	**RT/DAS**	63.9	38.6 ± 5.8	25.3 ± 0.9
8.	**CCCP/DAS**	61.7	34.8 ± 5.7	26.8 ± 5.4
9.	**RT/3-BP**	54.8	31.6 ± 3.7	23.2 ± 1.5
10.	**IACS-010759/DAS**	51.0	32.1 ± 4.0	18.9 ± 5.0
11.	**RT/LND**	49.1	21.5 ± 3.2	27.6 ± 8.8
12.	**ABT-199/LND**	47.8	15.9 ± 7.2	31.9 ± 14.8
13.	**ABT-199/2-DG**	44.8	25.6 ± 7.6	19.2 ± 3.9
14.	**RT/VIN**	42.4	21.9 ± 7.6	20.6 ± 1.4
15.	**ET/LND**	42.0	14.9 ± 3.5	27.1 ± 2.4
16.	**RT/MID**	42.0	22.5 ± 3.3	19.5 ± 4.0
17.	**ET/DAS**	41.6	22.1 ± 3.0	19.5 ± 3.3
18.	ET/MID^b^	39.5	6.7 ± 4.6	32.8 ± 3.9
19.	**IACS-010759/MID**	38.1	26.8 ± 7.2	11.3 ± 2.4
20.	**ara-C/MID**	38.0	13.1 ± 1.5	24.9 ± 1.6
21.	**CCCP/2-DG**	37.3	23.5 ± 5.7	13.8 ± 2.7
22.	**CCCP/3-BP**	34.6	23.6 ± 3.1	11.0 ± 0.7
23.	**IACS-010759/2-DG**	32.9	19.9 ± 7.8	13.0 ± 3.1
24.	ET/VIN^b^	32.2	7.4 ± 4.0	24.7 ± 0.6
25.	ET/2-DG	31.2	21.4 ± 2.2	9.8 ± 2.8
26.	ara-C/VIN^b^	30.3	5.7 ± 1.9	24.5 ± 2.0
27.	IACS-010759/LND^b^	29.0	23.1 ± 7.7	5.9 ± 2.3
28.	ara-C/3-BP^b^	27.6	22.4 ± 1.5	5.1 ± 1.0
29.	ara-C/2-DG	25.7	17.8 ± 3.9	7.9 ± 1.5
30.	ara-C/LND	22.9	17.7 ± 6.9	5.1 ± 1.2
31.	ara-C/DAS	19.3	8.6 ± 1.3	10.7 ± 0.4
32.	IACS-010759/3-BP	19.0	8.8 ± 2.0	10.2 ± 3.2
33.	ET/3-BP	12.4	7.4 ± 2.8	5.1 ± 3.3
34.	CCCP/VIN	3.5	1.8 ± 1.8	1.7 ± 1.2
35.	ABT-199/MID^c^	2.1	2.1 ± 2.1	0 ± 0
36.	CCCP/MID^c^	0.0	0 ± 0	0 ± 0

a *Based on at least 3 independent biological replicates*.

b *Drug combinations (n = 5) with opposite profile in MOLM-13 and OCI-AML2 cell line: maximal synergy higher than 20 in one cell line and lower than 10 in another one*.

c *Drug combinations (n = 2), antagonistic in one or both AML cell lines*.

*Combinations (n = 22) fitting in the cutoff for testing on normal blood cells (average value of maximal synergy is equal to or higher than 20 in at least one cell line and equal to or higher than 10 in both cell lines) are in bold*.

### Identification of Selective Compound Combinations

Twenty-two drug combinations showed promising synergy in cytotoxicity. However, it remained possible that they would have the same effect on healthy cells. Therefore, we tested the same drug combinations at the same doses in peripheral blood mononuclear cells (PBMCs) isolated from blood donations from healthy volunteers. These data were then used to build synergy landscapes for each combination. Compounds that also showed strong synergy in PBMCs were excluded from further analysis, as they were unlikely to be selective for tumor cells, and it would be difficult to find therapeutic indices that would be amenable for patient treatment.

**Figure 3:**
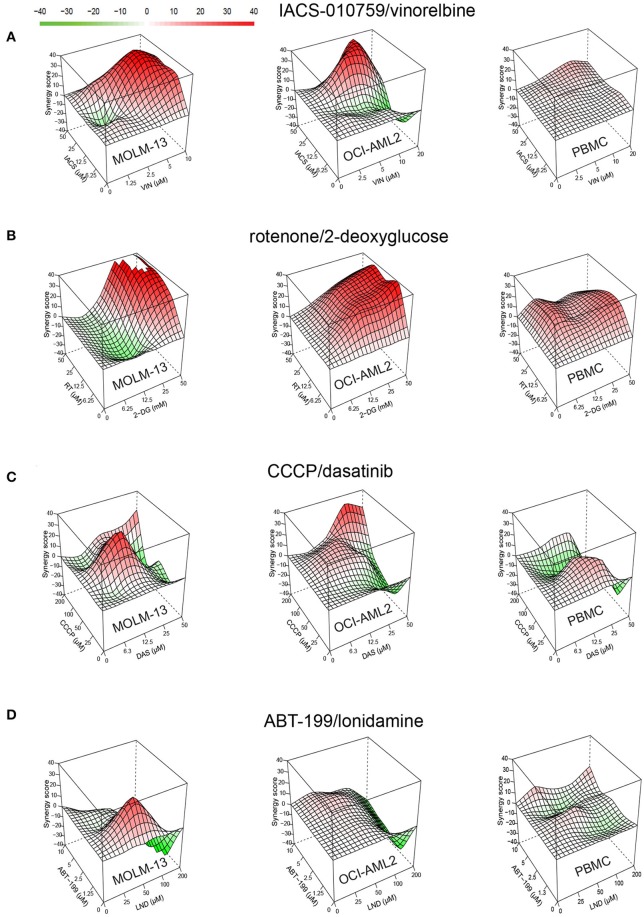
Drug combinations demonstrate higher synergy in AML than in healthy cells. Synergy landscapes for MOLM-13, OCI-AML2, or healthy PBMCs after treatment with **(A)** IACS-010759/vinorelbine, **(B)** rotenone/2-deoxy-D-glucose, **(C)** CCCP/dasatinib, and **(D)** ABT-199/lonidamine. Drug combination landscapes: *z-axis*, synergy score (ranges from −40 in green to +40 in red); *x/y-axes*, drug1/drug2 concentration range, respectively. Drug combination landscapes were built using Bioconductor package “synergyfinder.” One representative replicate (with maximal synergy closest to the average value of three biological replicates) is shown.

Cytotoxicity was then ranked by determining the difference between the survival of PBMCs and each of the AML cell lines. Overall, 21 of the 22 compounds showed some level of selectivity in at least one of the concentrations tested, supporting our approach. Four drug combinations showed at least a 50% reduction in survival between AML cells and healthy PBMCs in at least one dosage: IACS-010759 (ETC Complex I inhibitor) with vinorelbine (a microtubule destabilizing agent), which had an 80.3% difference; rotenone (ETC Complex I poison) and 2-deoxy-D-glucose (a glycolytic inhibitor), which demonstrated a 56.2% difference, CCCP (an ionophore that disrupts the mitochondrial membrane potential) and dasatinib (a tyrosine kinase inhibitor), which displayed 55.1% less killing in PBMCs, and ABT-199 (venetoclax, an anti-apoptotic inhibitor) and lonidamine (a glycolytic inhibitor), which showed 50.7% reduced cytotoxicity (Table 2, Figure S3, Table S7). For all of these combinations, synergy in healthy PBMCs was markedly lower than in AML cells (Figure 3).

**Table 2:** Effective mitocan-based drug combinations ranked in order of decreasing selectivity.

**Rank**	**Drug combination**	**Difference in survival between PBMC and MOLM-13, %**	**Difference in survival between PBMC and OCI-AML2, %**	**Average difference in survival^a^, MOLM-13, OCI-AML2, %**	**Doses of drug1/drug2, corresponding to maximal selectivity**
1.	**IACS/VIN**	80.0	80.5	**80.3**	IACS 25 μM/VIN 10 μM
2.	**RT/2-DG**	45.8	66.6	**56.2**	RT 50 μM/2-DG 50 mM
3.	**CCCP/DAS**	59.7	50.5	**55.1**	CCCP 200 μM/DAS 50 μM
4.	**ABT-199/LND**	51.5	49.8	**50.7**	ABT-199 1.3 μM/LND 50 μM
5.	IACS/2-DG	38.3	53.6	45.9	IACS 50 μM/2-DG 50 mM
6.	RT/VIN	75.7	15.9	45.8	RT 6.3 μM/VIN 5 μM
7.	RT/MID	51.5	32.6	42.0	RT 12.5 μM/MID 1.3 μM
8.	CCCP/2-DG	55.8	16.9	36.4	CCCP 200 μM/2-DG 50 mM
9.	ABT-199/3-BP	41.9	29.6	35.8	ABT-199 1.3 μM/3-BP 12.5 μM
10.	ET/LND	13.5	56.5	35.0	ET 25 μM/LND 100 μM
11.	RT/DAS	33.4	35.5	34.5	RT 12.5 μM/DAS 50 μM
12.	Ara-C/MID	23.3	44.4	33.8	Ara-C 50 μM/MID 10 μM
13.	IACS/DAS	25.2	41.9	33.5	IACS 25 μM/DAS 50 μM
14.	ET/DAS	32.9	31.4	32.2	ET 50 μM/DAS 50 μM
15.	ABT-199/VIN	20.0	40.7	30.3	ABT-199 10 μM/VIN 10 μM
16.	CCCP/LND	23.8	26.4	25.1	CCCP 200 μM/LND 200 μM
17.	RT/LND	36.1	12.5	24.3	RT 12.5 μM/LND 50 μM
18.	ABT-199/3-BP	19.9	23.0	21.4	ABT-199 10 μM/3-BP 5 μM
19.	IACS/MID	27.1	13.8	20.5	IACS 12.5 μM/MID 2.5 μM
20.	ABT-199/2-DG	22.6	8.4	15.5	ABT-199 1.3 μM/2-DG 25 mM
21.	CCCP/3-BP	13.3	7.0	10.1	CCCP 200 μM/3-BP 5 μM
22.	RT/3-BP	NS; Not selective

a *Maximal difference in survival between normal PBMC and AML (average for MOLM-13 and OCI-AML2 cells), %. Data from all biological replicates (n = 3) were combined*.

*Combinations chosen for further studies (average maximal difference in survival >50%) are in bold*.

*NS, non-significant difference in survival between normal PBMC and AML (MOLM-13/OCI-AML2) cells*.

*Not selective, significantly lower survival of normal PBMCs (not selective combination)*.

**Figure 4:**
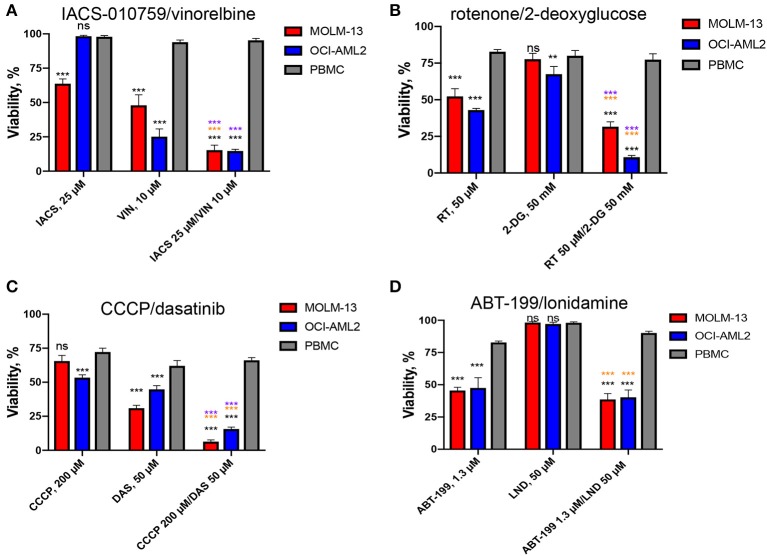
Effect of top selective drug combinations and corresponding single drugs on AML cell lines and healthy PBMCs. Survival of MOLM-13, OCI-AML2, or healthy PBMCs following single and combinatorial treatments with dosages corresponding to the maximal average difference in survival of AML vs. PBMCs. **(A)** IACS-010759/vinorelbine, **(B)** rotenone/2-deoxy-D-glucose, **(C)** CCCP/dasatinib, **(D)** ABT-199/lonidamine. Shown are the mean from at least three independent biological replicates (mean ± SEM). Significance of changes in survival was assessed via Student's *t*-test. ^***^p < 0.001; ^**^p < 0.01; ns: *p* > 0.05. Black stars or ns indicate comparison of AML cells vs. healthy PBMCs under the same treatment condition; purple stars indicate significantly lower survival under combinatorial treatment compared to single mitocan for each cell line; orange stars indicate significantly lower survival under combinatorial treatment compared to single complementary drug for each cell line.

For most treatments, tumor cell survival was significantly reduced after exposure to treatment with a combination of drugs, compared to treatment with them individually. This was particularly evident when one or both cell lines were resistant to one of the drugs. For example, OCI-AML2 was resistant to IACS-010759 used alone, showing essentially no effect from treatment at 25 μM (Figure 4). When combined with 10 μM vinorelbine, the synergistic effect dropped survival to ~15%. Lonidamine was ineffective at 50 μM against either AML cell line, but showed a synergistic effect when combined with ABT-199 at 1.3 μM. Similarly, 50 mM 2-deoxyglucose had essentially the same effect on MOLM-13 cells and PBMCs (Figure 4), but the addition of 50 μM rotenone strongly increased cell death in the former, but left the latter intact.

All but one of the remaining 18 combinations were synergistic and selective, but had a difference <50%, ranging from 10.1 to 45.9% (Table 2, Figures S4–S13). Interestingly, the combination of IACS-010759 and 2-deoxy-D-glucose, which targets complex I and glycolysis much like rotenone and 2-deoxyglucose, fell just short of our cutoff for selectivity, with the difference in death between AML cells and PBMCs of 45.9%, suggesting that targeting these two pathways may prove to be a particularly effective strategy (Table S7).

**Figure 5:**
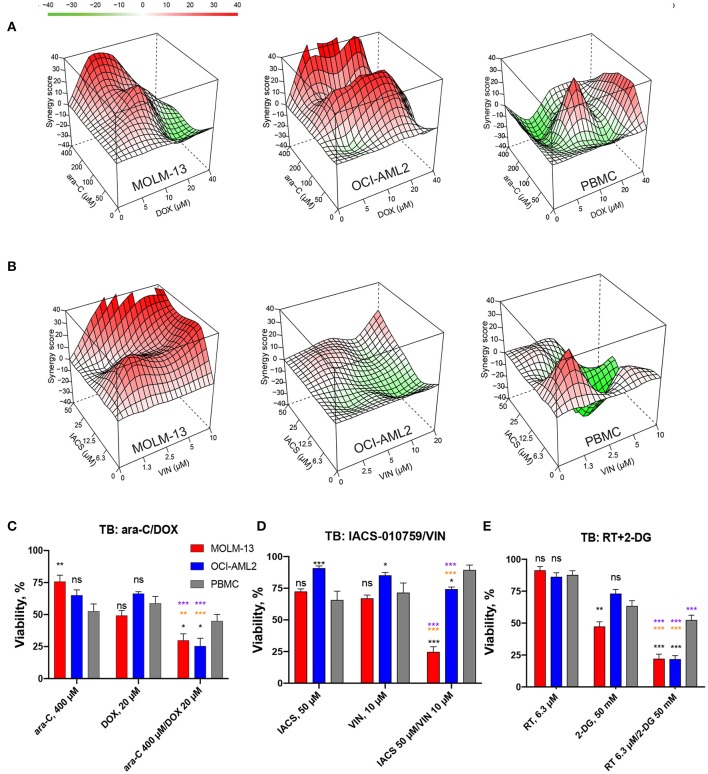
Comparison of cytotoxic effect and selectivity of IACS-010759/vinorelbine and rotenone/2-deoxy-D-glucose drug pairs with cytarabine/doxorubicin by trypan blue exclusion. **(A,B)** Drug combination landscapes for AML cell lines or PBMCs were built using Bioconductor package “synergyfinder.” One representative replicate (with maximal synergy closest to its average value) is shown: **(A)** cytarabine/doxorubicin, **(B)** IACS-010759/vinorelbine. **(C–E)** Survival of MOLM-13, OCI-AML2, or healthy PBMCs following single or combinatorial treatments with doses of maximal selectivity: **(C)** cytarabine/doxorubicin, **(D)** IACS-010759/vinorelbine, **(E)** rotenone/2-deoxy-D-glucose. Shown are mean ± SEM from three independent biological replicates. Significance of changes in survival were assessed via Student's *t*-test. ^***^p < 0.001; ^**^p < 0.01; ^*^p < 0.05; ns: *p* > 0.05. Black stars or ns indicate comparison of AML cells vs. healthy PBMCs under the same treatment condition; purple stars indicate significantly lower survival under combinatorial treatment compared to single mitocan for each cell line; orange stars indicate significantly lower survival under combinatorial treatment compared to single complementary drug for each cell line.

Finally, we compared efficiency and selectivity of IACS-010759/vinorelbine and rotenone/2-deoxy-D-glucose, the two treatments with the greatest synergy and efficacy, with those of the compounds from induction and consolidation therapy, cytarabine (ara-C)/doxorubicin (DOX). Cytotoxicity was evaluated using trypan blue (TB) exclusion. MTC doses were determined for cytarabine and doxorubicin as described above for the screening compounds and were used to build combination landscapes (Figures 5A,B, Figure S14A). Our tests revealed conditions where ara-C/DOX, IACS/VIN or RT/2-DG combinations resulted in significantly lower viability in both AML cell lines compared to healthy PBMCs (Figures 5C–E). The maximum difference in survival between AML and PBMCs was 17.4% for ara-C/DOX, 40% for IACS-010759/vinorelbine, and 31% for RT/2-DG. The difference in survival between healthy and AML cells for IACS/VIN or RT/2-DG, as determined via trypan blue exclusion, was lower than observed via Hoechst/PI staining. One possible explanation for this difference is that propidium iodide is more accurate at determining cell viability than trypan blue exclusion. This has previously been demonstrated for human hematopoietic stem cells (40).

### Selective Cytotoxicity of IACS-010759/Vinorelbine Combination May Be Associated With Early ATP Loss in AML Cells

**Figure 6:**
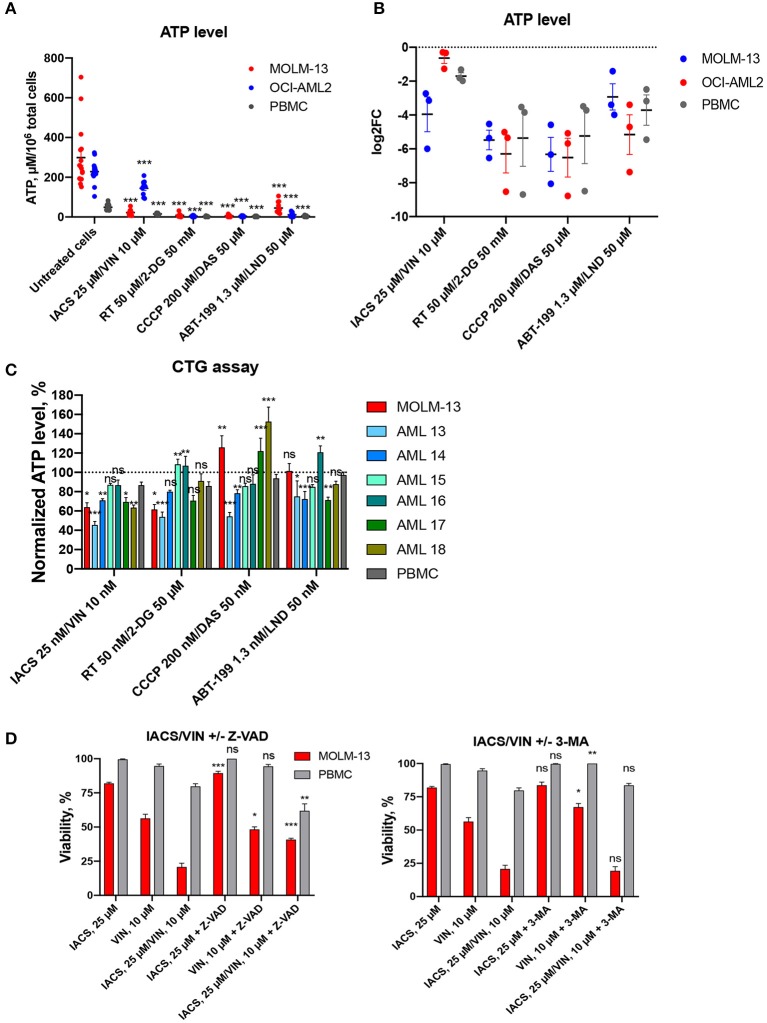
Treatment-induced changes in ATP level and activation of cell death. Absolute **(A)** or relative **(B)** ATP levels following 16 h treatment with DMSO or four selected drug combinations (IACS-010759/vinorelbine, rotenone/2-deoxy-D-glucose, ABT-199/lonidamine, or CCCP/dasatinib). Shown are mean ± SEM from at least three independent biological replicates. Significance of changes in ATP in treated vs. untreated cells for each cell line or PBMCs was assessed via Student's *t*-test. ^***^p < 0.001. **(C)** ATP levels, obtained with CellTiter-Glo^R^ assay and normalized to DMSO control (shown as *y* = 100), following treatment for 2 h with 1/1,000 doses corresponding to maximal selectivity of drug combinations in AML cells (MOLM-13 or primary AML samples), or healthy PBMCs. Shown are the means from 1 to 3 independent biological replicates (mean ± SEM). Significance of difference in AML vs. PBMCs was assessed via Student's *t*-test. **(D)** Changes in cellular viability after IACS-010759/vinorelbine or corresponding single drug treatment with or without addition of cell death inhibitors: Z-VAD, 40 μM, left; 3-MA, 5 mM, right. Shown are the means from three independent biological replicates (mean ± SEM). Significance of difference in survival with and without inhibitor was assessed via Student's *t*-test. ^***^p < 0.001; ^**^p < 0.01; ^*^p < 0.05; ns: *p* > 0.05.

Since all four of the combinations included a mitochondria-targeting drug, and two included glycolytic inhibitors as well, we tested whether these drug combinations' function was mediated by disrupting intracellular ATP production. First, each cell line was treated with the drug combination that induced the highest difference in survival for 16 h. ATP levels were then measured and normalized to the total cell number. While we observed a significant decrease in ATP level in all cell lines tested, the amount of the decrease did not differ between leukemia and normal cells, preventing differentiation on this basis (Figures 6A,B). After 16 h treatment, we observed profound ATP depletion, ranging from 1.2 to 4-fold decrease after treatment with IACS-010759/vinorelbine in OCI-AML2 cells and PBMCs up to 100-fold decrease after CCCP/dasatinib treatment in all cell lines tested.

Next, we measured the effect of each of the eight compounds that comprised the four selected drug combinations. For these experiments, each cell line was treated with single compounds and ATP measurements were collected (Figure S15). Again, we observed similar changes in AML cells and PBMCs: significant decrease under most of combinational treatments compared to single drugs. Interestingly, neither IACS-010759 nor vinorelbine alone reduced ATP levels in leukemia cell lines compared to untreated condition.

We also treated AML cells (MOLM-13 and six primary AML samples) and healthy PBMCs with a highly sensitive ATP detection reagent (Cell-Titer Glo^R^, CTG). Cells were pre-treated for a shorter period of time (2 h instead of 16) and at a lower concentration (0.001 dose of maximum selectivity), and then ATP levels were measured (Figure 6C). Contrary to the results observed after a longer period of time and with more compound, these assays illustrated a difference between AML cells (MOLM-13, 4 primary AML samples out of 6) compared to PBMCs after treatment with the combination of IACS-010759 and vinorelbine. For the other three selected combinations, we observed different responses in primary AML cells: either significant decrease/increase, or no changes in normalized ATP level compared to PBMCs. Surprisingly, treatment with CCCP/dasatinib combination for 2 h enhanced ATP production in MOLM-13 cells and several patient samples (*n* = 2/6). The diverse response to treatment suggests that the drug combinations may have disparate mechanisms for cytotoxicity.

To gain additional insight into the cell death phenomenon, we tested the ability of two compounds that compromise cell death pathways—the pan-caspase inhibitor Z-VAD-FMK to inhibit apotosis and 3-methyladenine (3-MA) to limit autophagy. MOLM-13 AML cells and PBMCs were each treated with 25 μM IACS-010759 and 10 μM vinorelbine (the combination showing the highest specificity) as well as 40 μM Z-VAD-FMK, 5 mM 3-MA, or DMSO (Figure 6D). Cells were also treated with individual compounds. After treatment, cell viability was assayed using the Hoechst/PI co-staining method. Z-VAD-FMK, the pan-caspase inhibitor, significantly reduced the cytotoxicity seen in MOLM-13 cells exposed to IACS-010759 or the combination of drugs. This suggests that IACS-010759 activates a mitochondria-dependent apoptotic cell death pathway in leukemia cells. This is consistent with reports that rotenone, which also inhibits ETC Complex I, also induces apoptosis (41). In that case, rotenone induces apoptosis through enhancing mitochondrial ROS production.

### IACS-010759/Vinorelbine and Rotenone/2-deoxy-D-Glucose Combinations Change Mitochondrial Bioenergetic Parameters

**Figure 7:**
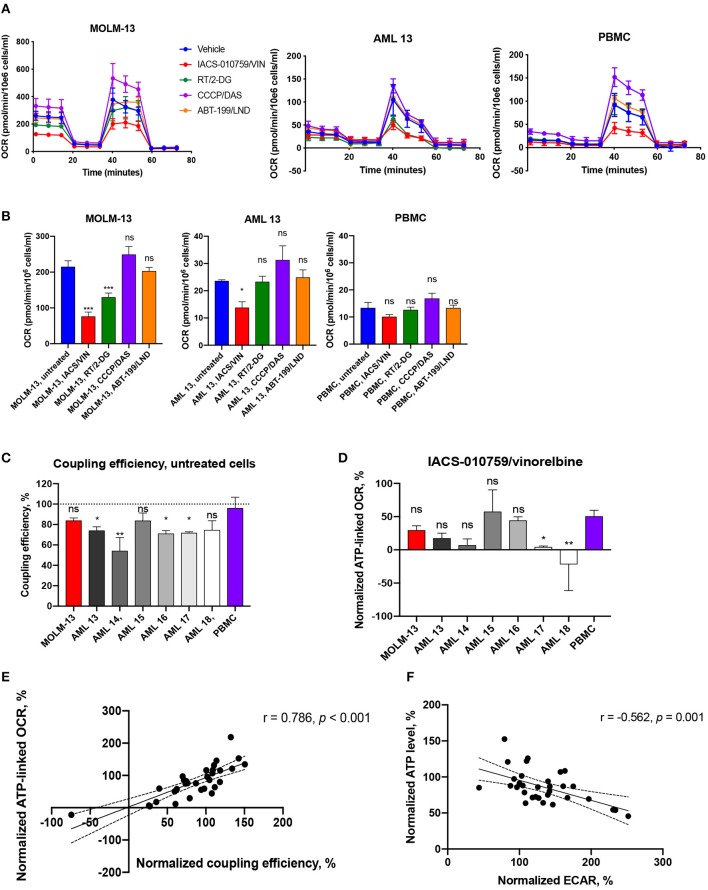
Bioenergetic profiling of immortalized and primary AML cells and healthy PBMCs after treatment with selected drug combinations. **(A)** Oxygen consumption rate (OCR) was measured using a Seahorse flux analyzer. OCR measured in cells either untreated (blue) or treated with IACS-010759 25 nM/vinorelbine 10 nM (red), rotenone 50 nM/2-deoxy-D-glucose 50 μM (green), CCCP 200 nM/dasatinib 50 nM (purple), or ABT-199 1.3 nM/lonidamine 50 nM (orange) for 2 h. One representative replicate of time-course OCR measurements (mean ± SD) in MOLM-13 cells, representative primary AML sample (AML 13), and healthy PBMCs is shown. See Figure S16 for additional primary cell data. **(B)** Treatment-induced changes in basal mitochondrial respiration in AML cells (MOLM-13, representative AML sample) or healthy PBMCs. Shown is mean ± SEM. Significance of difference between treated and untreated cells was assessed via ANOVA with subsequent pairwise comparisons. **(C)** Coupling efficiency of untreated AML cells or healthy PBMCs. Shown is mean ± SEM. Significance of difference vs. PBMCs was assessed via ANOVA with subsequent pairwise comparisons. **(D)** ATP-linked respiration after IACS/VIN treatment in a panel of AML cells or PBMCs, normalized to DMSO control. Significance of difference vs. PBMCs was assessed via ANOVA with subsequent pairwise comparisons. **(E)** Significant positive correlation between normalized coupling efficiency, %, and normalized ATP-linked respiration, %, across all studied samples under four selected treatments. **(F)** Significant negative correlation between normalized levels of ATP, %, and ECAR, %, across all studied samples under four selected treatments. Mito-stress test was repeated three times independently for MOLM-13 cells and PBMCs, and once for each primary AML sample. ^***^p < 0.001; ^**^p < 0.01; ^*^p < 0.05; ns: *p* > 0.05. Correlations were assessed using Pearson *r* coefficient, line of best fit represents linear regression line.

We also used a Seahorse metabolic flux analyzer to evaluate mitochondrial function in AML cell lines and primary AML cells (*n* = 6) that were treated with the four selective drug combinations. Cells were treated at 1/1,000 dose of their maximal selectivity to minimize cell death: IACS-010759 25 nM/vinorelbine 10 nM, rotenone 50 nM/2-DG 50 μM, CCCP 200 nM/dasatinib 50 nM, or ABT-199 1.3 nM/lonidamine 50 nM, for 2 h (Figure 7, Table S8, Figure S16).

As we observed with the Cell-Titer Glo assay, we observed disparate outcomes when cells were treated with compounds prior to the measurement of mitochondrial bioenergetics. For example, the combination of ABT-199 and lonidamine increased maximal OCR (oxygen consumption rate) and, by extension, spare capacity, by 29–35% in patient sample AML16. However, the same parameters were decreased by 39% in another primary cell sample, AML15 (Table S8). Neither MOLM-13 cells nor other primary AML samples demonstrated significant changes in mitochondrial respiration after ABT-199/LND treatment.

The most active combination in terms of inhibition of mitochondrial function was IACS-010759 and vinorelbine. It decreased basal, maximal, and ATP-linked OCR by 35–122%, as well as reduced spare capacity by 40–88% and coupling efficiency by 21–175% in MOLM-13 cells and the majority of patient AML samples. However, most of these mitochondrial parameters were also decreased after treatment with IACS/VIN in normal PBMCs. The most significant exception was basal respiration, which was more dramatically affected in MOLM-13 cells (Figure 7B, Table S8). Interestingly, as Figure 7C shows, untreated PBMCs were estimated to have significantly better coupling efficiency than most untreated AML samples (*n* = 4/6), which is in line with our previously published work (24). This fact likely contributes to the selectivity of the drugs and drug combinations that reduce coupling efficiency, such as IACS/VIN or RT/VIN. In support of this hypothesis, the average ratio of coupling efficiency (IACS+VIN/untreated cells) between all studied AML cells was 0.38, but the same ratio in PBMCs was 0.6. Similarly, the average ratio of ATP-linked respiration was 0.2 in AML cells vs. 0.5 in normal PBMCs (Figure 7D).

Another combination, rotenone/2-deoxyglucose, did not significantly affect healthy PBMCs, but reduced basal OCR (MOLM-13 cells), maximal OCR (MOLM-13 cells, primary AML, *n* = 2/6), ATP-linked OCR (MOLM-13, primary AML, *n* = 1/6), and spare capacity (primary AML, *n* = 1/6) in some of the cases. Interestingly, both IACS/VIN and RT/2-DG combinations significantly enhanced ECAR (extracellular acidification rate) by 27–234% in MOLM-13 cells and studied patient samples (*n* = 5/6), as well as in PBMCs in case of IACS/VIN (Table S8). Previous reports have shown that inhibiting Complex I of the ETC with IACS-010759 or rotenone upregulates glucose consumption and glycolysis (42, 43). As expected, normalized coupling efficiency was also significantly associated (*r* = 0.786, *p* < 0.001) with ATP-linked OCR (Figure 7E). This is also consistent with our observations that normalized ATP level inversely correlated with normalized ECAR values (*r* = −0.562, *p* = 0.001) suggesting that ATP depletion might play a signaling role in activating glycolysis (Figure 7F).

### Identified Drug Combinations Have Strong Cytotoxic Synergy Against Other Types of Leukemia

**Figure 8:**
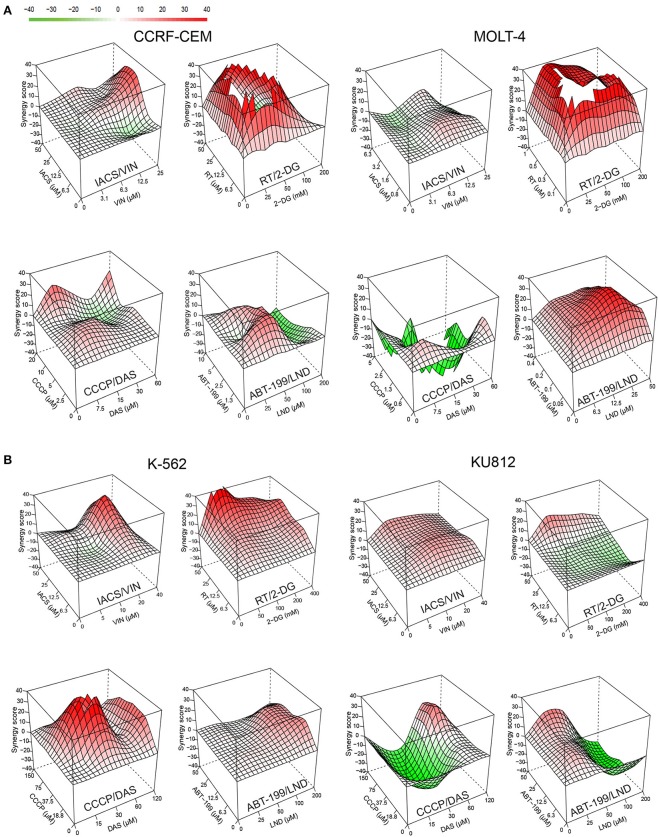
Effect of selected mitocan-based drug combinations on other types of leukemia. **(A,B)** Synergy landscapes for ALL, **(A)** (CCRF-CEM cells, left, or MOLT-4 cells, right) or CML, **(B)** (K-562 cells, left, or KU812 cells, right) treated with IACS-010759/vinorelbine, rotenone/2-deoxy-D-glucose, CCCP/dasatinib, or ABT-199/lonidamine. Drug combination landscapes: *z-axis*, synergy score (ranges from −40, green, to +40, red); *x/y-axes*, drug1/drug2 concentration range, respectively. Drug combination landscapes were built using Bioconductor package “synergyfinder.” One representative replicate (with maximal synergy closest to the average value of three biological replicates) is shown. For specific drug names and drug concentrations, refer to Table S4.

To determine whether the four best combinations have broader activity against other leukemias, we tested their effects against additional hematological malignancies. Acute lymphoblastic leukemia (ALL, CCRF-CEM and MOLT-4) and chronic myeloid leukemia (CML, K-562 and KU812) cells were treated using the same strategy as AML cell lines (i.e., MTCs were determined for each compound/cell line combination, and then synergy testing was performed). Promisingly, all of the combinations demonstrated synergistic interactions, although to different extents. Rotenone/2-deoxy-D-glucose displayed the highest sum of maximum synergies for the ALL and CML cells (Figure 8, Table 3).

**Table 3:** Effect of selected mitocan combinations on acute lymphoblastic leukemia (ALL) and chronic myelogenous leukemia (CML) cell lines.

**Cell line (leukemia type)**	**Maximal synergy^a^, mean ± SEM**			
	IACS/VIN^*^	RT/2-DG^**^	CCCP/DAS^*^	ABT-199/LND^**^
CCRF-CEM (ALL)	30.8 ± 8.2	84.9 ± 1.8	21.5 ± 2.7	21.1 ± 3.5
MOLT-4 (ALL)	9.0 ± 2.0	62.8 ± 10.9	8.1 ± 1.3	44.1 ± 5.0
K-562 (CML)	32.7 ± 7.1	53.2 ± 14.4	53.1 ± 3.9	17.4 ± 4.7
KU812 (CML)	13.0 ± 2.7	17.7 ± 2.4	21.6 ± 5.7	20.5 ± 2.4
Sum	85.5	218.6	104.3	103.1

a *Based on three independent biological replicates*.

* *Combinations that have been tested for toxicity against normal blood cells at doses corresponding to CML treatment (maximal synergy cutoff >10 in both CML cell lines)*.

** *Combinations that have been tested for toxicity against normal blood cells at doses corresponding to both ALL and CML treatment (maximal synergy cutoff >10 in both ALL/CML cell lines)*.

**Figure 9:**
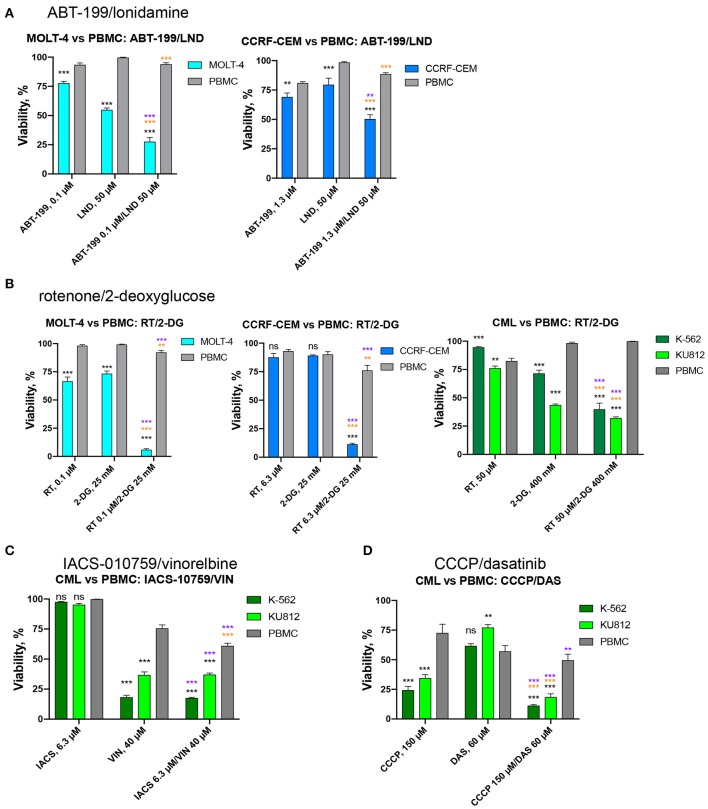
Effect of selected drug combinations and corresponding single drugs on ALL/CML cell lines or healthy PBMCs. Survival of CCRF-CEM or MOLT-4 cells (ALL), K-562 and KU812 cells (CML), or healthy PBMCs following single and combinatorial treatments at the dose of maximum selectivity. **(A)** ABT-199/lonidamine against ALL cells vs. PBMCs; **(B)** rotenone/2-deoxy-D-glucose against ALL/CML cells vs. PBMCs. **(C,D)** IACS-010759/vinorelbine or CCCP/dasatinib against CML cells vs. PBMCs. Shown are mean ± SEM from at least three independent biological replicates. Significance of changes in survival was assessed via Student's *t*-test. ^***^p < 0.001; ^**^p < 0.01; ns: *p* > 0.05. Black stars or ns indicate comparison of ALL/CML cells vs. healthy PBMCs under the same treatment condition; purple stars indicate significantly lower survival under combinatorial treatment compared to single mitocan for each cell line; orange stars indicate significantly lower survival under combinatorial treatment compared to single complementary drug for each cell line.

Next, we chose those combinations from the four tested that had synergy >10 in both cell lines of the same leukemia type, and >20 in one of them (Table 3) and treated healthy PBMCs with the same doses of drugs. This allowed us to successfully identify conditions under which normal blood cells survived significantly better than leukemia cells. It is worth noting that, while only two combinations out of four met these selection criteria for ALL (RT/2-DG and ABT-199/LND), the difference in survival between cancerous cells and healthy PBMCs was more profound for ALL than CML (Figure 9). Four drug combinations demonstrated 33.7–86.2% lower killing of PBMCs than ALL and CML cells. Only one combination, ABT-199/lonidamine, did not appear to be selective for CML treatment. These observations highlight the potential for these drug combinations to be effective against multiple leukemia types.

### Identified Drug Combinations Retain Their Synergy Against Primary AML Samples

**Figure 10:**
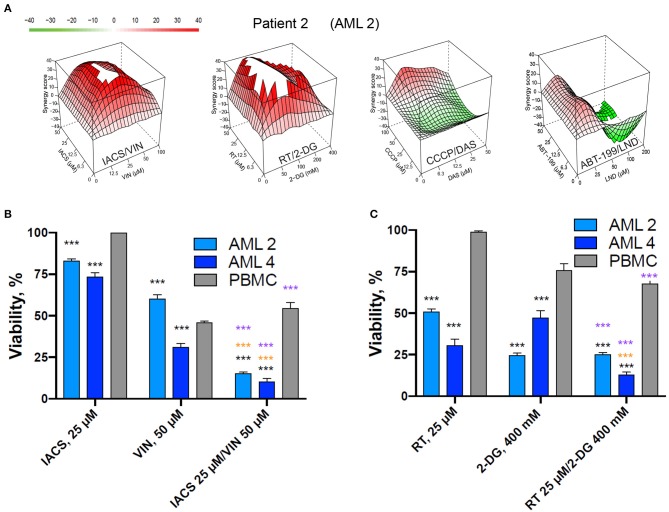
Selected mitocan-based drug combinations retain their synergy in primary AML samples. **(A)** Synergy landscapes for representative patient sample treated with IACS-010759/vinorelbine, rotenone/2-deoxy-D-glucose, CCCP/dasatinib, or ABT-199/lonidamine. Drug combination landscapes: *z-axis*, synergy score (ranges from −40, green, to +40, red); *x/y-axes*, drug1/drug2 concentration range, respectively. For drug1/drug2 concentration ranges refer to Table S5. Drug combination landscapes were built using Bioconductor package “synergyfinder.” One representative replicate (with maximal synergy closest to the average value of three biological replicates) is shown. **(B,C)** The effect of two selected drug combinations (IACS-010759/vinorelbine or rotenone/2-deoxy-D-glucose) with highest average synergy and corresponding single drugs on primary AML samples or healthy PBMCs. Shown are mean ± SEM from three independent biological replicates. Significance of changes in survival was assessed via Student's *t*-test. ^***^p < 0.001. Black stars indicate comparison of primary AML cells vs. healthy PBMCs under the same treatment condition; purple stars indicate significantly lower survival under combinatorial treatment compared to single mitocan for each cell type; orange stars indicate significantly lower survival under combinatorial treatment compared to single complementary drug for each cell type.

To determine whether these four combinations (IACS-010759/vinorelbine, rotenone/2-deoxyglucose, CCCP/dasatinib, and ABT-199/lonidamine) have therapeutic lead potential, they were tested in a tertiary screen against primary AML cells derived from patients (*n* = 12) using the process described above. Two of the four drug combinations (IACS-010759/vinorelbine and rotenone/2-deoxy-D-glucose), exhibited synergy scores higher than 10 in every patient tested (except the case of IACS/VIN in sample AML11), with average maximum synergy across all patients reaching 31.4 and 66.1, respectively (see Table 4 for individual patient results and Figure 10 for an example patient). Thus, they met our original criteria for the identification of synergistic combinations in cell lines. The combination of CCCP and dasatinib displayed synergy in 10 of 12 patients (83.3%), ABT-199/lonidamine was synergistic in 8 of 12 patients (66.7%). Given the data available, it is difficult to explain these variations. One possibility is that subtle differences in genotype or metabolism are affecting the level of sensitivity.

**Table 4:** Effect of selected mitocan combinations on primary AML samples.

**Patient sample**	**Maximal synergy^a^, mean ± SEM**			
	**IACS/VIN**	**RT/2-DG**	**CCCP/DAS**	**ABT-199/LND**
AML 1	49.2 ± 18.6	54.7 ± 7.8	27.4 ± 2.8	2.3 ± 0.5
AML 2	50.7 ± 3.2	73.8 ± 3.7	25.0 ± 1.0	17.7 ± 1.1
AML 3	27.0 ± 6.9	50.5 ± 2.6	14.8 ± 4.0	26.9 ± 1.8
AML 4	20.1 ± 9.2	79.7 ± 5.6	7.1 ± 4.2	13.8 ± 11.4
AML 5	10.1	54.3	0	47.7
AML 6	42.1 ± 2.7	81.3 ± 1.0	28.6 ± 3.8	10.5 ± 3.9
AML 7	16.5 ± 8.2	81.5 ± 7.7	11.2 ± 0.8	17.4 ± 4.3
AML 8	40.7 ± 5.7	57.9 ± 2.2	12.9 ± 2.5	7.5 ± 7.5
AML 9	18.3 ± 4.6	78.9 ± 2.4	29.2 ± 15.2	8.9 ± 2.7
AML 10	50.9	78.1	56.2	15.0
AML 11	4.6 ± 0.1	25.6 ± 3.9	33.1 ± 23.0	16.2 ± 11.7
AML 12	46.5 ± 8.9	76.7 ± 6.1	17.1 ± 6.9	0 ± 0
Average	31.4	66.1	21.9	15.3

a *Based on 1–3 independent biological replicates*.

Interestingly, there was a strong correlation (*R* = 0.909, data in Table 5) between synergy scores in AML cell lines and primary AML cells. Although this correlation was not statistically significant (*p*-value = 0.091), that is most likely because the number of combinations tested was small. Regardless, the strength of this correlation between immortalized and primary AML cells is promising and suggests that this approach may prove useful for identifying promising treatment options.

**Table 5:** Comparison of the effect of selected mitocan combinations on AML cell lines, primary AML samples, and healthy PBMCs.

**Drug combination**	**Mean synergy coefficient**		
	**AML cell lines^a^**	**Primary AML samples^b^**	**Normal PBMCs^c^**
IACS/VIN	34.4	31.4	4.9
RT/2-DG	39.9	66.1	−8.0
CCCP/DAS	30.8	21.9	−19.3
ABT-199/LND	23.9	15.3	−6.7

a *Shown is the mean of maximal synergy between MOLM-13 and OCI-AML2 cell lines*.

b *Shown is the mean of maximal synergy between all analyzed primary AML samples (n = 12)*.

c *Shown is the mean synergy in PBMCs corresponding to the dose resulting in maximal difference in survival AML vs. PBMC (for doses, see Table 2)*.

## Discussion

Most cancers, including leukemias, have long been considered to be almost entirely dependent on glycolysis for their energy needs. Consequently, glycolytic inhibitors have been tested as monotherapies in these cells, and have often worked, although not always with great efficiency (44). Contrasting this conventional wisdom, a growing role for mitochondrial OxPhos in these cells is beginning to be recognized (45, 46). In a previous study, we discovered that leukemia cells are particularly sensitive to treatments that target mitochondria, likely due to poor mitochondrial coupling (24). We have noted that this effect was exacerbated by combining the mitochondrial uncoupler CCCP with 2-deoxy-D-glucose, a glycolytic inhibitor. In this study, we tested whether a wider variety of mitocans and other classes of chemotherapeutics also exhibit synergistic cytotoxicity. We identified four drug combinations, IACS-010759/vinorelbine, rotenone/2-deoxy-D-glucose, CCCP/dasatinib, and ABT-199/lonidamine, that had synergetic and selective cytotoxicity in AML cells. Importantly, all of the combinations also exhibited synergistic killing of either ALL, CML, or both, suggesting a wider utility than previously anticipated.

Consistent with our previous report, two out of the four combinations (ABT-199/lonidamine and rotenone/2-deoxy-D-glucose) combined glycolytic inhibitors with mitocans. Interestingly, ABT-199 is a Bcl-2 inhibitor and rotenone is a poison that inactivates complex I of the electron transport chain. Similarly, the Zweidler-McKay lab reported that sub-therapeutic doses of the electron transport chain complex III inhibitor antimycin A combined with propyl 3-bromo-2-oxopropanoate, a third-generation glycolytic inhibitor, effectively killed leukemia cells through severe ATP depletion (47). These data argue that a broad cross-section of mitochondrial function can effectively be targeted in this way, and opens the question of which combinations will be most effective in which types of cancer.

To the best of our knowledge, the combination of IACS-010759 and microtubule inhibitor vinorelbine has not yet been studied in relation to leukemia treatment. However, vinorelbine-based chemotherapy has been reported as effective against aggressive therapy-refractory leukemias, including AML and CML (48, 49). IACS-010759, which is currently in clinical development, is a novel OxPhos inhibitor that targets mitochondrial complex I (35). This compound caused only minor cell death of chronic lymphocytic leukemia at 24 h of treatment, but the addition of 2-deoxy-D-glucose significantly increased cytotoxicity (43). Here we demonstrate that the combination of IACS-010759 and vinorelbine impairs several mitochondrial functions, such as oxygen consumption and coupling efficiency. The treatment also significantly inhibited basal mitochondrial respiration, seemingly with some specificity for AML cells. The already low coupling efficiency of AML cells may be contributing to the selectivity of the cytotoxicity observed for the IACS-010759/vinorelbine combination and the loss of ATP induced by this treatment.

Rotenone, which also compromises complex I, was one of the more effective mitocans. All rotenone-containing combinations had strong synergetic effects on leukemia cells. Unfortunately, it was also generally quite toxic to PBMCs and is well-known to have strong acute neuronal toxicity and induces parkinsonism (50). It also causes bone marrow depletion, hematopoietic suppression, and bone atrophy (51). These facts may ultimately preclude its use as a safe anti-cancer compound, but further investigation is necessary to test this.

It is worth noting that this screen was carried out with the intention of finding treatments that were effective against a variety of AML types. For this reason, we used two genomically-distinct AML cell lines: MOLM-13 (*FLT3-ITD, CBL*ΔExon8, MLL-AF9 fusion) and OCI-AML2 (DNMT3A R882C), and successful combinations needed to exhibit substantial synergy in both cell lines and increased efficacy against these lines compared to PBMCs. This is the likeliest explanation for why IACS-010759 and rotenone, which share the same target, were not functionally interchangeable. For example, while the combination of IACS-010759 and vinorelbine appeared on our final list of specific and effective treatments, the combination of rotenone and vinorelbine did not. This absence is easily explained, however, by the preselected cutoff criteria. The combination of rotenone and vinorelbine was very selective against MOLM-13 cells but had little selectivity against OCI-AML2 cells. It also likely explains why CCCP, which uncouples the electron transport chain from ATP synthesis, only appears in one of our hits. Our previous data suggested a correlation between mitochondrial coupling efficiency and treatment efficacy (24). Based on this, we expected CCCP to effectively combine with other treatments. However, as shown by the differential nuclear staining assay, the combination of CCCP and 2-deoxy-D-glucose was less effective against OCI-AML2 cells than against MOLM-13, and its combination with either lonidamine or with 3-bromopyruvate lacked selectivity for tumor cells. Since AML can be caused by a variety of genetic lesions, cancers may have significant differences in drug sensitivity. This was one of the key reasons drug combinations were screened against more than one cell line and validated in primary AML cells, to identify combinations with the greatest likelihood of efficacy across different AML subtypes. This screening method necessarily carried a risk of false negatives, but we accepted this possibility, since it is simple to rescreen individual combinations on particular cell lines of interest.

Tumor resistance is a major problem associated with chemotherapy (52). One method to combat this is to use two or more drugs in combination. Combination therapy is rapidly becoming the standard of care in a variety of cancers because it has several advantages, including limiting the development of resistance and potential drug synergies that may allow reduced drug concentrations to be used (53, 54). Repurposing existing drugs in new combinations could provide new possibilities with reduced cost and time for development, while potentially minimizing side effects by lowering drug dosages (6). Mitochondrial dependency of leukemia cells and their altered oxidative metabolism have already been noticed as frequent abnormality existing in various AML subgroups (55, 56). Moreover, several drug combinations based on mitotoxic drugs, such as cytarabine and mitoxantrone or cytarabine with anthracyclines (doxorubicin, etc.), have already demonstrated effectiveness in AML treatment, including clinical trials and standard of care therapy (57, 58). AML resistance is frequently linked to the existence of a subpopulation of transformed pluripotent hematopoietic stem cells that divide very slowly (generally known as leukemia stem cells, or LSCs) (59). The slow division rate of these cells makes them relatively resistant to anti-cancer chemotherapeutics that interfere with cell division. A wide variety of drugs used to treat cancers target actively proliferating cells (e.g., taxanes like paclitaxel or docetaxel, or cyclin-dependent kinase inhibitors like abemaciclib or palbociclib), making them less effective against LSCs. Importantly, LSCs, like other leukemia cells appear to be more dependent upon mitochondria for energy production than most cancer cells (60–62), and thus may be more sensitive to the drug cocktails identified here. This hypothesis is currently under active investigation.

## Data Availability Statement

All datasets generated for this study are included in the article/Supplementary Material.

## Ethics Statement

The studies involving human participants were reviewed and approved by the Institutional Review Board for the University of Texas MD Anderson Cancer Center and the Institutional Review Board for Rice University. The patients/participants provided their written informed consent to participate in this study.

## Author Contributions

SP, JP, and NK designed the experiments. SP, JP, and NB performed the experiments. NB and MK provided the patient samples. SP wrote the first draft. SP, JP, NB, MK, and NK edited the manuscript. NK overall project design, supervision, and funding acquisition.

### Conflict of Interest

The authors declare that the research was conducted in the absence of any commercial or financial relationships that could be construed as a potential conflict of interest.

## Abbreviations

ara-C: cytarabine
CCCP: carbonyl cyanide *m*-chlorophenylhydrazone
DAS: dasatinib
ECAR: extracellular acidification rate
ET: etoposide
ETC: electron transport chain
IACS: IACS-010759
LND: lonidamine
MID: midostaurin
OCR: oxygen consumption rate
OxPhos: oxidative phosphorylation
RT: rotenone
VIN: vinorelbine
2-DG: 2-deoxy-D-glucose
3-BP: 3-bromopyruvate.
