# Supplementary material for: Utilizing Synergistic Potential of Mitochondria-Targeting Drugs for Leukemia Therapy
Source: Front Oncol. 2020 Apr 3;10:435. doi: 10.3389/fonc.2020.00435 (PMC7146088; doi:10.3389/fonc.2020.00435)
Supplement: Supplementary file 1 [file Table_1.pdf]

Supplementary Material

Supplementary Tables

Table S1. List of cell lines included in the study.

Cell line	Cell line origin	Disease	Age	Sex	Ploidy	Genetic mutations / rearrangements
OCI-AML2	Peripheral blood	Acute myeloid leukemia	65	M	Hyperdiploid 48(43-49)	*DNMT3A* [PMID: 21904384]
MOLM-13	Peripheral blood	Acute myeloid leukemia	20	M	Hyperdiploid 51(48-52)	FLT3-ITD[1], CBL deltaExon8 mutant, *MLL-AF9* fusion [PMID: 12529668; 9305600]
K-562	Bone marrow	Chronic myelogenous leukemia	53	F	Triploid	*CDKN2A, TP53* [PMID 17088437], BCR/ABL fusion
KU812	Peripheral blood	Chronic myelogenous leukemia	38	M	Polyploid<br><br>58	Ph1 (Philadelphia) chromosome [PMID 3858609]
CCRF-CEM	Peripheral blood	Acute lymphoblastic leukemia	4	F	Aneuploid (modal number = 47; range = 41 to 95)	*CDKN2A, TP53, KRAS, PTEN, FLT3* [PMID 17088437]
MOLT-4	Peripheral blood	Acute lymphoblastic leukemia	19	M	Hypertetra-ploid (modal chromosome number - 95 in 24% of cells)	*CDKN2A, TP53, NRAS, PTEN* [PMID 17088437]

[1] internal tandem duplication

Table S2. Characteristics of patients whose AML samples were used in the study.

N	age	sex	WBC	% blast	Pretreatment	Mutation status: gene mutations/ genetic rearrangements	Karyotype
1.	52	F	8.7	84%	Nivolumab Azacitidine Ipilimumab	NF1; SUZ12; TP53	41,XX,-4,add(5)(q11.2), add(6)(p25),-7,+8, der(11)dup(11)(q13q25)dup (11)(q25q13),-12,-17,-18,-21[7] /41~42,idem,add(13)(q34),-14,+mar[cp12]/51,XX,+3,add(5)(q11.2),+6,add(6)(p25)x2,-7,+8,+8,+10,+11,der(11)dup(11)(q13q25)dup(11)(q25q13),-12,add(13)(q34),+14,+15,-18,-21[1]
2.	36	F	22.1	14%	Cytarabine Hydroxyurea	ASXL, FLT3, NRAS, RUNX1, PHF6	46, XX
3.	66	M	18.6	70%	Hydroxyurea	PML/RARA	46, XY
4.	48	F	13.4	97%	Decitabine Venetoclax Quizartinib	BCOR,PM1,FLT3, WT1	46, XX
5.	64	M	8.7	39%	AMG 330 Hydroxyurea	CREBBP, PHF6, RUNX1, TET2	46,XY,t(7;10)(q32;q22)[18]/46,XY[2]
6.	70	M	11.2	16%	Hydroxyurea, Cytarabine	SRFS2	46, XY
7.	53	F	22.7	89%	Hydroxyurea, 2014-0057 Hu8F4	FLT3, TET2	48,XX,+8,inv(16)(p13.1q22),+22[19]/48,idem,t(12;22)(p13;q11.2)[1]
8.	30	M	2.4	76%	Azacitidine Hydroxyurea	EED, GATA2, PTPN11, RUNX1	45,XY,t(3;3)(q21;q26.2),del(6)(q12),-7[20]
9.	25	M	44.4	96%	Hydroxyurea, Pentamidine, Fludarabine, IDArubicin with CYTarabine plus Venetoclax	PTPN11	46,XY,+8,-16,der(21)t(16;21)(p11.2;q22)[7]/46,idem,add(1)(p36.1),add(4)(q21),t(4;8)(p15.2;q13),del(17)(p11.2),add(18)(q23),+mar[cp11]//46,XX[2]
10.	64	M	18.2	62%	Cytarabine	NRAS, PRPF40B, PTPN11	46,XY,t(6;11)(q27;23)[18]/44~46,idem,+1,der(1;15)(q10;q10)[cp2]
11.	74	F	8.3	94%	IMGN632 and AzaCITIDine, Hydroxyurea, azaCITIDine, Avelumab, and Venetoclax	GATA1, RAD21, TP53	44,XX,dic(5;17)(q11.2;p11.2),add(7)(p12),add(8)(p12),dic(13;14)(p13;p12),-19,+mar[16]/43~45,idem,+mar[cp3]/46,XX[1]
12.	33	M	22.5	98%	PLX51107 + azaCITIDine	BCOR, ETV6, GATA2, IKZF1, NRAS, SF3B1, WT1	45,X,-Y,inv(3)(q21q26.2)[1]/45,idem,del(6)(q21q25),add(20)(q11.2)[18]/45,X,-Y,der(1)t(1;3)(q42;p21),der(3) t(1;3)inv(3)(q21q26.2)[1]
13.	61	M	5.9	82%	None	TP53, ZRSR2	45,XY,del(5)(q15q33),-7,add(10)(q24),+11,-14,-16,+add(17)(q25),del(17)(p11.1),del(17)(p

								11.2),-18,+21,add(22)(q13)[11]/46,XY[9]
14.	74	M	4.9	14%	DAUNOrubicin-CYTarabine liposomal (CPX-351) + Gemtuzumab	ASXL, IDH1, NRAS, RUNX1, SRSF2, STAG2, TERT, TET2	47,XY,+8[17]/46,XY[3
15.	54	F	25.8	87%	Fludarabine Cytarabine	DNMT3A, NF1, TET2, TP53	42,XX,del(5)(q13q33),-7,-14,-17,-18[2]/41~42,idem,-X,del(3)(q26),-5,del(6)(q13q23),-11,add(11)(q25),add(12)(p11.2),-15,-16,add(16)(q24),add(19)(q12),del(20)(q11.2q13.1),+22,+1~5mar[cp17]//46,XY[1]
16.	82	M	66.8	100%	Hydroxyurea<br><br>Azacitidine	CBL, EXH2, JAK2, SF3B1, SMC3, TET2	46,XY[20]
17.	74	F	9.3	73%	IMGN632 and AzaCITIDine, azaCITIDine, Avelumab, and Venetoclax	GATA1, RAD21, TP53	44,XX,dic(5;17)(q11.2;p11.2),add(7)(p12),add(8)(p12),dic(13;14)(p13;p12),-19,+mar[16]/43~45,idem,+mar[cp3]/46,XX[1]
18.	76	M	1.7	69%	None<br><br>Past: VEN + IMGN	KRAS, TP53	38~47<2n>,XY,del(5)(q13),-7,+8,+11,add(11)(p15),-16,der(16)t(1;16)(p13;q13),-18,+19,-22,+mar[cp18]/46,XY[2]

Table S3. Drugs used for screening of mitocan-based combinations against AML cell lines.

Mitocans	Tyrosine Kinase Inhibitors	Anti-microtubule	Anti-glycolytic
OXPHOS inhibitors: rotenone[2], IACS-010759[1]	Midostaurin[1]<br>Dasatinib[1]	Vinorelbine[1,2]	2-deoxy-D-glucose (2-DG)[2]<br>3-bromopyruvate (3-BP)[2]<br>Lonidamine[2]
DNA-targeted: cytarabine[1], etoposide[1]			
Pro-apoptotic: ABT-199[1]			
Uncouplers: carbonyl cyanide *m*-chlorophenylhydrazone (CCCP)[2]			

[1] Drugs being used in regimens against AML/considered as promising by clinical trials;
[2] Drugs having selectivity against leukemia cells compared to normal blood cells as defined by preliminary cytotoxicity assays.

Table S4. Concentrations of drugs used for building combination landscapes in cell lines*.

Drug	MOLM-13	OCI-AML2	CCRF-CEM	MOLT-4	K-562	KU812
Rotenone (RT), μM	0, 6.3, 12.5, 25, 50[1]	0, 6.3, 12.5, 25, 50[1]	0, 6.3, 12.5, 25, 50[1]	0, 0.1, 0.25, 0.5, 1[1]	0, 6.3, 12.5, 25, 50[2]	0, 6.3, 12.5, 25, 50[2]
IACS-010759, μM	0, 6.3, 12.5, 25, 50[3]	0, 6.3, 12.5, 25, 50[3]	0, 6.3, 12.5, 25, 50[3]	0, 0.8, 1.6, 3.2, 6.3[1]	0, 6.3, 12.5, 25, 50[3]	0, 6.3, 12.5, 25, 50[3]
Cytarabine (ara-C), μM	0, 50, 100, 200, 400[3]	0, 50, 100, 200, 400[3]	N/A[4]	N/A	N/A	N/A
Etoposide (ET), μM	0, 25, 50, 100, 200[3]	0, 12.5, 25, 50, 100[1]	N/A	N/A	N/A	N/A
ABT-199, μM	0, 1.3, 2.5, 5, 10[1]	0, 1.3, 2.5, 5, 10[1]	0, 1.3, 2.5, 5, 10[1]	0, 0.05, 0.1, 0.2, 0.4[1]	0, 6.3, 12.5, 25, 50[2]	0, 6.3, 12.5, 25, 50[2]
CCCP, μM	0, 25, 50, 100, 200[1]	0, 25, 50, 100, 200[1]	0, 2.5, 5, 10, 20[1]	0, 0.6, 1.3, 2.5, 5[1]	0, 18.8, 37.5, 75, 150[1]	0, 18.8, 37.5, 75, 150[1]
Midostaurin (MID), μM	0, 1.3, 2.5, 5, 10[2]	0, 1.3, 2.5, 5, 10[2]	N/A	N/A	N/A	N/A
Dasatinib (DAS), μM	0, 6.3, 12.5, 25, 50[1]	0, 6.3, 12.5, 25, 50[1]	0, 7.5, 15, 30, 60[1]	0, 7.5, 15, 30, 60[1]	0, 15, 30, 60, 120[1]	0, 15, 30, 60, 120[1]
Vinorelbine (VIN), μM	0, 1.3, 2.5, 5, 10[1]	0, 2.5, 5, 10, 20[1]	0, 3.1, 6.3, 12.5, 25[1]	0, 3.1, 6.3, 12.5, 25[1]	0, 5, 10, 20, 40[1]	0, 5, 10, 20, 40[1]
2-deoxy-D-glucose (2-DG), mM	0, 6.3, 12.5, 25, 50[1]	0, 6.3, 12.5, 25, 50[1]	0, 25, 50, 100, 200[1]	0, 25, 50, 100, 200[1]	0, 50, 100, 200, 400[2]	0, 50, 100, 200, 400[2]
3-bromopyruvate (3-BP), μM	0, 1.3, 2.5, 5, 10[1]	0, 2.5, 5, 10, 20[1]	N/A	N/A	N/A	N/A
Lonidamine (LND), μM	0, 25, 50, 100, 200[2]	0, 25, 50, 100, 200[2]	0, 25, 50, 100, 200[2]	0, 6.3, 12.5, 25, 50[1]	0, 25, 50, 100, 200[2]	0, 25, 50, 100, 200[2]

Maximal dose of single drug used for combination landscapes was defined by preliminary cytotoxicity assays:
[1]dose resulting at 30-50% survival for at least one AML cell line from two tested; 30-50% survival for ALL or CML cell line
[2]dose corresponding to the limit of drug (4x) solubility in test media
[3]dose corresponding to the limit of DMSO cytotoxicity
[4]N/A defines drugs that have not been used against ALL/CML cell lines

*The same range of 2-fold serial dilutions was used within same type of leukemia, e.g., 0, 0.8, 1.6, 3.2, 6.3 μM IACS-010759 for MOLT-4, and 0, 6.3, 12.5, 25, 50 μM IACS-010759 for CCRF-CEM.

Table S5. Concentrations of drugs used for building combination landscapes in primary AML cells.

Drug	AML 1	AML 2	AML 3	AML 4	AML 5	AML 6	AML 7	AML 8	AML 9	AML 10	AML 11	AML 12
IACS-010759, µM	0, 6.3, 12.5, 25, 50[3]	0, 6.3, 12.5, 25, 50[3]	0, 6.3, 12.5, 25, 50[3]	0, 6.3, 12.5, 25, 50[3]	0, 6.3, 12.5, 25, 50[3]	0, 6.3, 12.5, 25, 50[3]	0, 6.3, 12.5, 25, 50[3]	0, 3.1, 6.3, 12.5, 25[3]	0, 3.1, 6.3, 12.5, 25[3]	0, 3.1, 6.3, 12.5, 25[3]	0, 3.1, 6.3, 12.5, 25[3]	0, 3.1, 6.3, 12.5, 25[3]
VIN, µM	0, 6.3, 12.5, 25, 50[1]	0, 3.1, 6.3, 12.5, 25[1]	0, 12.5, 25, 50, 100[1]	0, 6.3, 12.5, 25, 50[1]	0, 3.1, 6.3, 12.5, 25[1]	0, 3.1, 6.3, 12.5, 25[1]	0, 3.1, 6.3, 12.5, 25[1]	0, 6.3, 12.5, 25, 50[1]	0, 12.5, 25, 50, 100[1]	0, 3.1, 6.3, 12.5, 25[1]	0, 6.3, 12.5, 25, 50[1]	0, 12.5, 25, 50, 100[1]
RT, µM	0, 6.3, 12.5, 25, 50[2]	0, 6.3, 12.5, 25, 50[2]	0, 6.3, 12.5, 25, 50[2]	0, 6.3, 12.5, 25, 50[2]	0, 6.3, 12.5, 25, 50[2]	0, 6.3, 12.5, 25, 50[2]	0, 6.3, 12.5, 25, 50[2]	0, 6.3, 12.5, 25, 50[2]	0, 6.3, 12.5, 25, 50[2]	0, 6.3, 12.5, 25, 50[2]	0, 6.3, 12.5, 25, 50[2]	0, 6.3, 12.5, 25, 50[2]
2-DG, mM	0, 37.5, 75, 150, 300[1]	0, 37.5, 75, 150, 300[1]	0, 50, 100, 200, 400[1,2]	0, 50, 100, 200, 400[2]	0, 50, 100, 200, 400[2]	0, 50, 100, 200, 400[2]	0, 50, 100, 200, 400[1,2]	0, 50, 100, 200, 400[1,2]	0, 50, 100, 200, 400[2]	0, 12.5, 25, 50, 100[1]	0, 50, 100, 200, 400[2]	0, 50, 100, 200, 400[1,2]
CCCP, µM	0, 6.3, 12.5, 25, 50[1]	0, 6.3, 12.5, 25, 50[1]	0, 12.5, 25, 50, 100[1]	0, 12.5, 25, 50, 100[1]	0, 3.1, 6.3, 12.5, 25[1]	0, 12.5, 25, 50, 100[1]	0, 6.3, 12.5, 25, 50[1]	0, 25, 50, 100, 200[1]	0, 25, 50, 100, 200[1]	0, 25, 50, 100, 200[1]	0, 25, 50, 100, 200[1]	0, 25, 50, 100, 200[1]
DAS, µM	0, 9.4, 18.8, 37.5, 75[1]	0, 9.4, 18.8, 37.5, 75[1]	0, 6.3, 12.5, 25, 50[1]	0, 12.5, 25, 50, 100[1]	0, 6.3, 12.5, 25, 50[1]	0, 6.3, 12.5, 25, 50[1]	0, 12.5, 25, 50, 100[1]	0, 6.3, 12.5, 25, 50[1]	0, 12.5, 25, 50, 100[1]	0, 6.3, 12.5, 25, 50[1]	0, 12.5, 25, 50, 100[1]	0, 6.3, 12.5, 25, 50[1]
ABT-199, µM	0, 6.3, 12.5, 25, 50[1,2]	0, 6.3, 12.5, 25, 50[1,2]	0, 6.3, 12.5, 25, 50[1,2]	0, 6.3, 12.5, 25, 50[1,2]	0, 1.6, 3.1, 6.3, 12.5[1]	0, 1.6, 3.1, 6.3, 12.5[1]	0, 0.8, 1.6, 3.2, 6.3[1]	0, 0.8, 1.6, 3.2, 6.3[1]	0, 6.3, 12.5, 25, 50[1,2]	0, 0.8, 1.6, 3.2, 6.3[1]	0, 6.3, 12.5, 25, 50[2]	0, 6.3, 12.5, 25, 50[2]
LND, µM	0, 25, 50, 100, 200[2]	0, 25, 50, 100, 200[2]	0, 25, 50, 100, 200[2]	0, 25, 50, 100, 200[2]	0, 25, 50, 100, 200[2]	0, 12.5, 25, 50, 100[1]	0, 25, 50, 100, 200[1,2]	0, 25, 50, 100, 200[2]	0, 12.5, 25, 50, 100[1]	0, 25, 50, 100, 200[2]	0, 25, 50, 100, 200[1,2]	0, 25, 50, 100, 200[1,2]

Maximal dose of single drug used for combination landscapes was defined by preliminary cytotoxicity assays:

[1]dose resulting at 30-50% survival

[2]dose corresponding to the limit of drug (4x) solubility in test media

[3]dose corresponding to the limit of DMSO cytotoxicity

Table S6. Example of calculation of maximal difference in survival: CCCP/dasatinib (CCCP/DAS) combination.

Dose[1]	Viability[2], MOLM-13, mean (1)	Viability[2], OCI-AML2, mean (2)	Viability[2], normal PBMC, mean (3)	Difference in survival, MOLM-13, (4)=(3)-(1)	Difference in survival, OCI-AML2, (5)=(3)-(2)	Average difference in survival, (6)=((4)+(5))/2,
CCCP 50 μM/DAS 25 μM	54.52%	66.98%	80.03%	25.50%	13.05%	19.28%
CCCP 100 μM/DAS 12.5 μM	64.40%	65.10%	78.40%	14.00%	13.30%	13.65%
CCCP 100 μM/DAS 25 μM	67.46%	68.37%	79.89%	12.43%	11.52%	11.98%
CCCP 200 μM/DAS 25 μM	25.44%	38.63%	60.07%	34.63%	21.44%	28.03%
CCCP 200 μM/DAS 50 μM	6.41%	15.59%	64.23%	57.82%	48.64%	53.23%[3]

[1]Considered were only doses at which viability of PBMCs was significantly higher than that of both AML cell lines;
[2]Based on at least 3 independent biological replicates;
[3]Maximal difference in survival is highlighted in red.

Table S7. Relationship between selectivity and synergy of 21 mitocan-based combinations.

Rank[1]	Dose of maximal selectivity	Difference PBMC - MOLM13, survival, %	Difference PBMC - OCI-AML2, survival, %	Average difference in survival, %[2]	Difference PBMC - MOLM13, synergy coefficient, arbitrary units	Difference PBMC - OCI-AML2, synergy coefficient, arbitrary units	Average difference in synergy, arbitrary units[3]
1.	IACS 25 µM/VIN 10 µM	80	80.5	**80.3**	-24.3	-25.8	-25.0
2.	RT 50 µM/2-DG 50 mM	45.8	66.6	**56.2**	-21.0	-29.4	-25.2
3.	CCCP 200 µM/DAS 50 µM	59.7	50.5	**55.1**	-37.2	-32.4	-34.8
4.	ABT-199 1.3 µM/LND 50 µM	51.5	49.8	**50.7**	-12.4	-10.8	-11.6
5.	IACS 50 µM/2-DG 50 mM	38.3	53.6	45.9	-6.0	-14.3	-10.1
6.	RT 6.3 µM/VIN 5 µM	75.7	15.9	45.8	-2.8	0.4	-1.2
7.	RT 12.5 µM/MID 1.3 µM	51.5	32.6	42	1.3	-6.4	-2.5
8.	CCCP 200 µM/2-DG 50 mM	55.8	16.9	36.4	-10.4	13.5	1.6
9.	ABT-199 1.3 µM/DAS 12.5 µM	41.9	29.6	35.8	-8.3	-19.6	-13.9
10.	ET 25 µM/LND 100 µM	13.5	56.5	35	-20.5	-41.5	-31.0
11.	RT 12.5 µM/DAS 50 µM	33.4	35.5	34.5	4.6	-7.4	-1.4
12.	Ara-C 50 µM/MID 10 µM	23.3	44.4	33.8	-8.7	-24.9	-16.8
13.	IACS 25 µM/DAS 50 µM	25.2	41.9	33.5	17.2	2.0	9.6
14.	ET 50 µM/DAS 50 µM	32.9	31.4	32.2	-1.3	-0.6	-1.0
15.	ABT-199 10 µM/VIN 10 µM	20	40.7	30.3	-9.3	-34.9	-22.1
16.	CCCP 200 µM/LND 200 µM	23.8	26.4	25.1	-34.3	-12.7	-23.5
17.	RT 12.5 µM/LND 50 µM	36.1	12.5	24.3	-0.9	-6.1	-3.5
18.	ABT-199 10 µM/3-BP 5 µM	19.9	23	21.4	-18.1	-35.9	-27.0
19.	IACS 12.5 µM/MID 2.5	27.1	13.8	20.5	13.8	-4.6	4.6

	μM						
20.	ABT-199 1.3 μM/2-DG 25 mM	22.6	8.4	15.5	-1.9	-2.6	-2.2
21.	CCCP 200 μM/3-BP 5 μM	13.3	7	10.1	-0.8	16.0	7.6

[1]Considered were only selective drug combinations (n = 21);
[2]Average between columns 3 and 4;
[3]Average between columns 6 and 7.

Table S8. Changes[*] in mitochondrial bioenergetic parameters after treatment with selected mitocan-based drug combinations (IACS-010759/vinorelbine, rotenone/2-DG, CCCP/dasatinib, ABT-199/lonidamine) in a panel of AML cells and normal PBMCs.

Drug combination	Bioenergetic parameter	MOLM-13	AML 13	AML 14	AML 15	AML 16	AML 17	AML 18	PBMC
IACS/ VIN	Basal OCR	***p* < 0.0001 -64%**	***p* = 0.0236 -35%**	NS	NS	***p* = 0.0004 -58%**	***p* < 0.0001 -77%**	NS	NS
	Maximal OCR	***p* < 0.0001 -64%**	***p* = 0.0014 -58%**	NS	***p* = 0.0161 -40%**	***p* < 0.0001 -71%**	***p* < 0.0002 -82%**	NS	***p* = 0.0001 -71%**
	ATP-linked OCR	***p* < 0.0001 -70%**	***p* = 0.0112 -82%**	NS	NS	***p* < 0.0008 -56%**	***p* = 0.0003 -93%**	***p* = 0.0055 -122%**	***p* = 0.0009 -49%**
	Proton leak	NS	NSNS	NS	NS	***p* = 0.0161 -29%**	NS	NS	NS
	Spare capacity	***p* = 0.0307 -63%**	***p* = 0.0062 -40%**	NS	NS	***p* < 0.0001 -73%**	***p* = 0.0116 -88%**	NS	***p* = 0.0001 -79%**
	Coupling efficiency	***p* = 0.0015 -21%**	***p* = 0.0134 -38%**	NS	NS	NS	***p* = 0.0005 -73%**	***p* = 0.0491 -175%**	***p* = 0.0009 -41%**
	ECAR	***p* = 0.0032 +33%**	***p* = 0.0008 +234%**	***p* = 0.0001 +138%**	***p* < 0.0001 +61%**	***p* < 0.0001 +75%**	***p* < 0.0001 +97%**	NS	***p* = 0.0341 +42%**
RT/ 2-DG	Basal OCR	***p* = 0.0003 -40%**	NS	NS	NS	NS	NS	NS	NS
	Maximal OCR	***p* = 0.0069 -36%**	***p* = 0.0203 -35%**	NS	NS	NS	***p* = 0.0454 -34%**	NS	NS
	ATP-linked OCR	***p* = 0.0011 -41%**	NSNS	NS	NS	NS	NS	***p* < 0.0288 -88%**	NS
	Proton leak	NS	NS	NS	NS	NS	NS	NS	NS
	Spare capacity	NS	***p* = 0.0245 -22%**	NS	NS	NS	NS	NS	NS
	Coupling efficiency	NS	NS	NS	NS	NS	NS	NS	NS
	ECAR	***p* < 0.0001 +47%**	***p* = 0.0019 +217%**	***p* = 0.0038 +113%**	***p* < 0.0001 +64%**	***p* < 0.0001 +57%**	***p* = 0.0097 +27%**	NS	NS
CCCP/ DAS	Basal OCR	NS	NS	NS	NS	NS	***p* = 0.0316 +22%**	NS	NS

	Maximal OCR	NS	**p = 0.0282<br>+37%**	NS	NS	NS	NS	NS	NS
	ATP-linked OCR	NS	NS	NS	NS	NS	NS	NS	NS
	Proton leak	NS	NS	NS	NS	NS	NS	NS	**p = 0.0308<br>+169%**
	Spare capacity	NS	NS	NS	NS	NS	NS	NS	NS
	Coupling efficiency	NS	NS	NS	NS	NS	NS	NS	**p = 0.0114<br>-28%**
	ECAR	NS	**p = 0.0023<br>+229%**	NS	NS	NS	NS	**p = 0.0047<br>-21%**	**p = 0.0457<br>+40%**
ABT-199/ LND	Basal OCR	NS	NS	NS	NS	NS	NS	NS	NS
	Maximal OCR	NS	NS	NS	**p = 0.0161<br>-39%**	**p = 0.0329<br>+29%**	NS	NS	NS
	ATP-linked OCR	NS	NS	NS	NS	NS	NS	NS	NS
	Proton leak	NS	NS	NS	NS	NS	NS	NS	NS
	Spare capacity	NS	NS	NS	**p = 0.048<br>-39%**	**p = 0.0235<br>+35%**	NS	NS	NS
	Coupling efficiency	NS	NS	NS	NS	NS	NS	NS	**p = 0.0135<br>-28%**
	ECAR	NS	NS	NS	**p < 0.0001<br>-43%**	NS	NS	NS	NS

*ANOVA with subsequent pairwise Fisher LSD test was used for group comparisons. Significant changes ($p < 0.05$) are in bold.
