# Supplementary material for: Utilizing Synergistic Potential of Mitochondria-Targeting Drugs for Leukemia Therapy
Source: Front Oncol. 2020 Apr 3;10:435. doi: 10.3389/fonc.2020.00435 (PMC7146088; doi:10.3389/fonc.2020.00435)
Supplement: Supplementary file 2 [file Data_Sheet_1.pdf]

Supplementary Figures

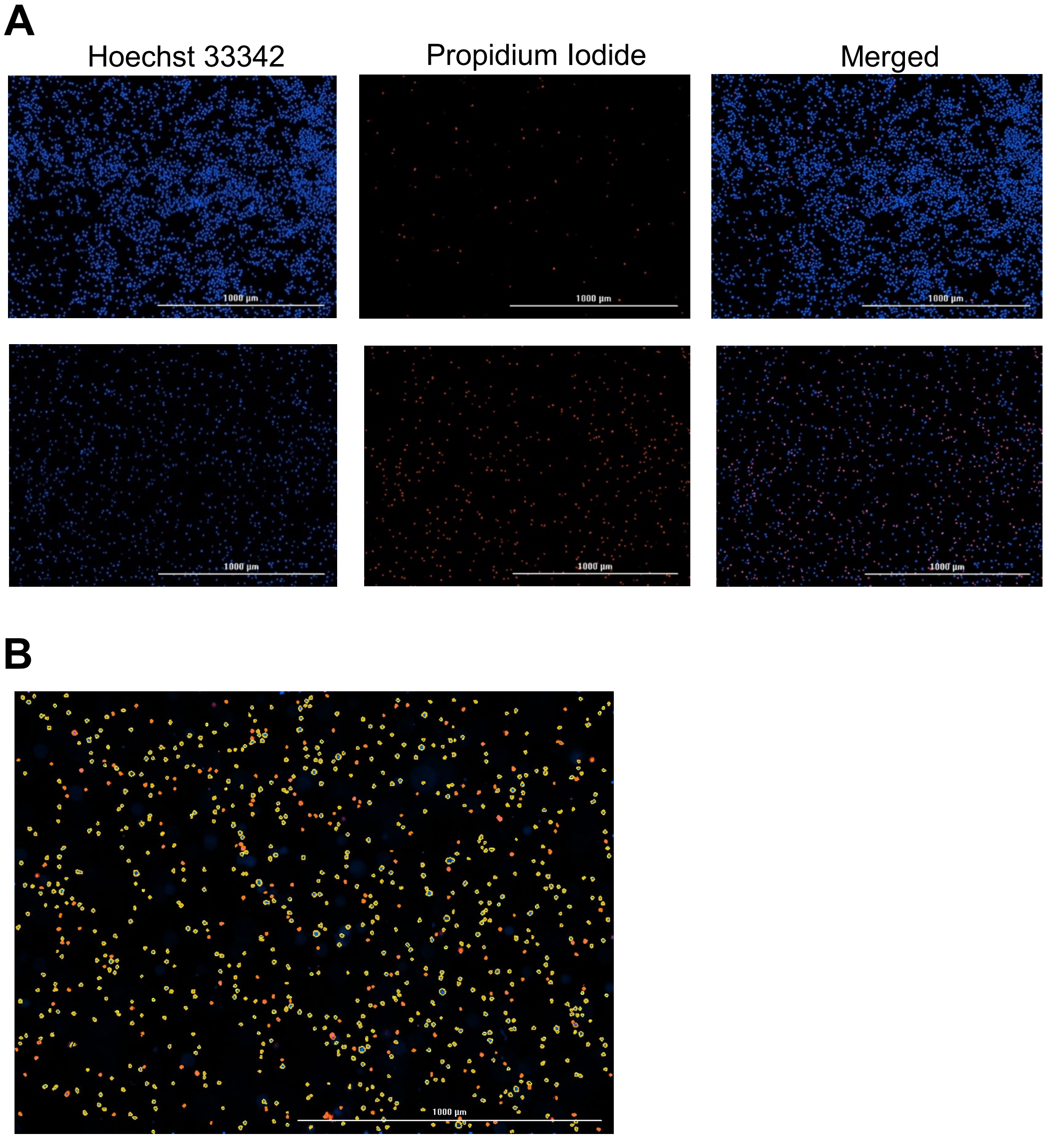

Figure S1. Example of cell viability assessment. (**A**) Fluorescent images of MOLM-13 cells treated either with DMSO (top panels) or 5 µM 3-bromopyruvate for 24 h. Images were acquired using DAPI (for Hoechst 33342) and Texas Red (for propidium iodide) filter sets for a Cytation5 multi-mode reader (Biotek). (**B**) Example of image analysis using Gen5 software. Nuclear mask (in yellow) represents Hoechst 33342-stained cell population (all cells). Dead cells double stained with PI (in red) were counted to determine survival rate. Scale bars represent 1 mm.

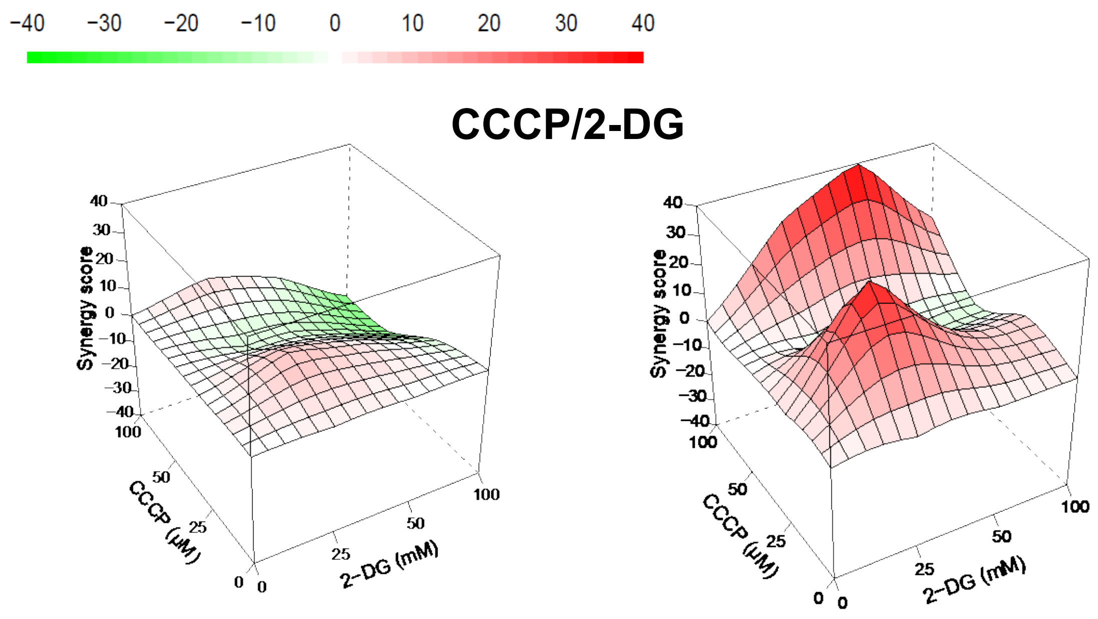

Figure S2. Optimization of a time point for combinatorial drug treatment experiments. Viability of MOLM-13 cells was measured at 14 h (left) or 24 h (right) after exposure to CCCP, 2-DG, or their combination at indicated concentrations. Drug combination landscapes were built using the Bioconductor package ‘synergyfinder’ using a Bliss model. 24 h treatment was chosen based on higher average and maximal synergy coefficients. One representative of three biological replicates is shown.

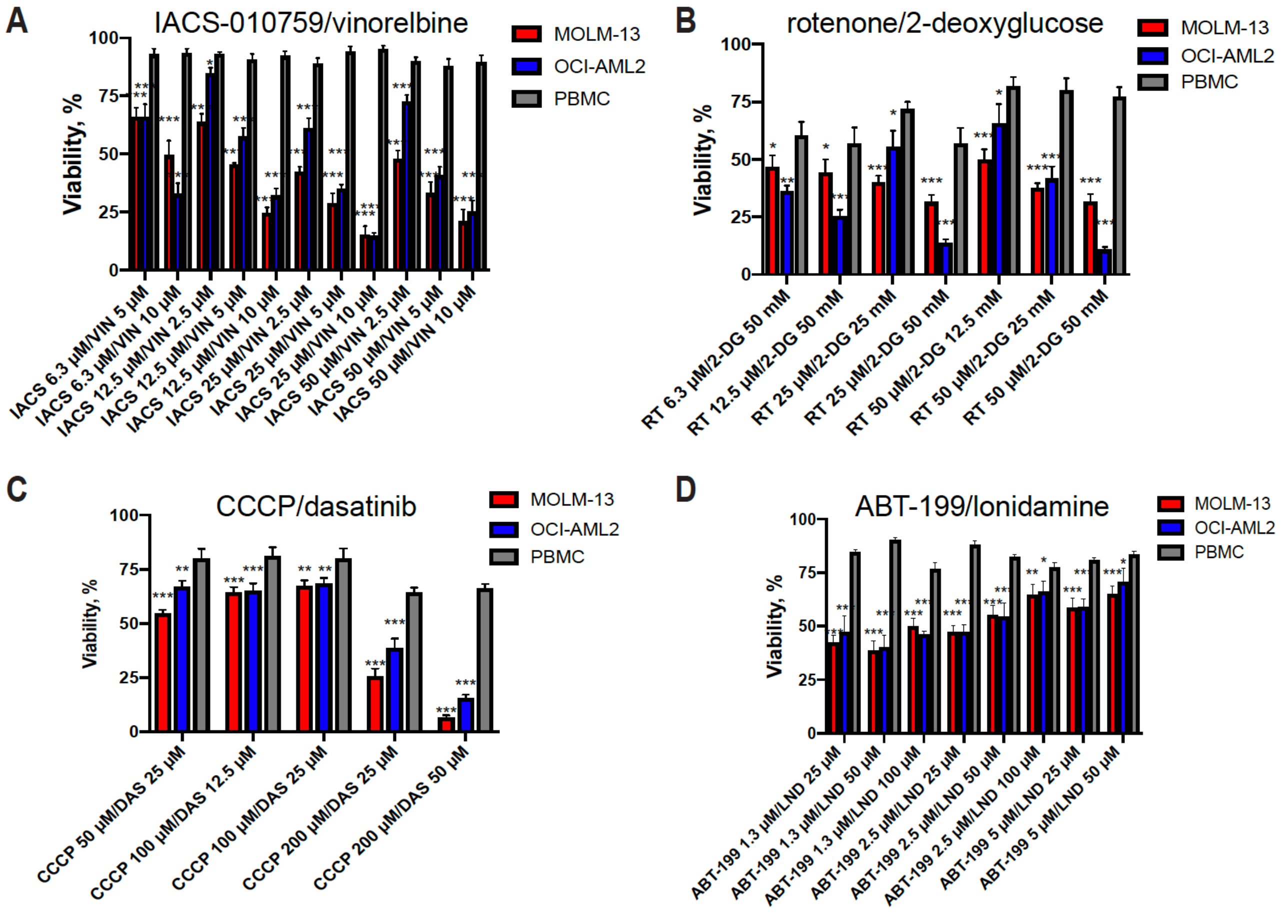

Figure S3. Selected combinations that preferentially kill AML cells. Survival of MOLM-13 and OCI-AML2 cells, or healthy PBMCs following treatment with (**A**) IACS-010759/vinorelbine, (**B**) rotenone/2-DG, (**C**) CCCP/dasatinib, or (**D**) ABT-199/lonidamine at specified concentrations. Only conditions where PBMC viability was significantly higher than that of both AML cell lines are shown. Bar plots represent the average survival of at least three independent biological replicates (mean ± SEM). Significance of difference in survival (AML cells vs. PBMCs) was assessed via Student's *t*-test. ***: $p < 0.001$; **: $p < 0.01$; *: $p < 0.05$.

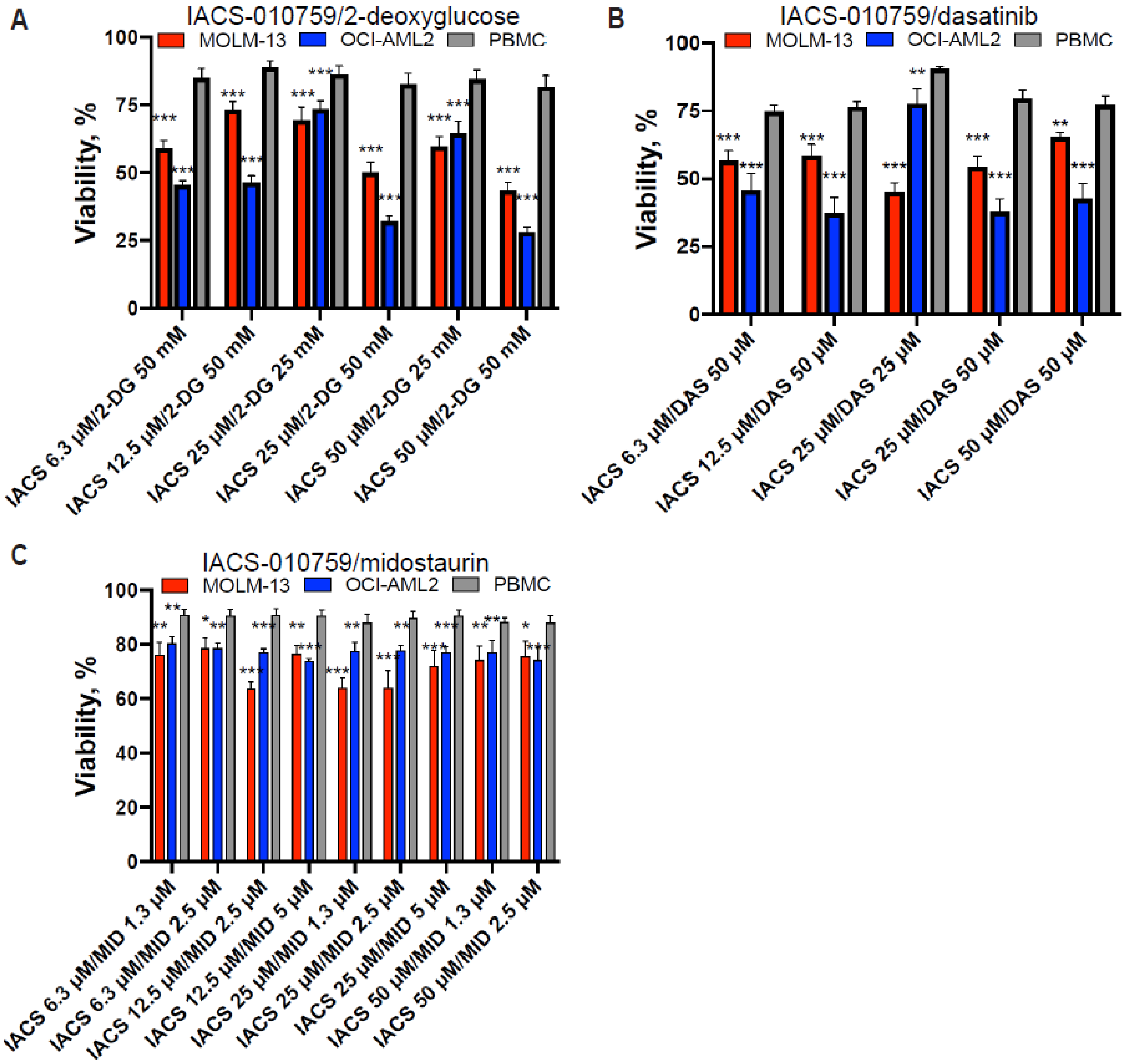

Figure S4. IACS-010759-based combinations display preferential selectivity toward AML cells. Survival of MOLM-13, OCI-AML2, or healthy PBMCs following treatment with (**A**) IACS-010759/2-deoxy-D-glucose, (**B**) IACS-010759/dasatinib, or (**C**) IACS-010759/midostaurin at specified concentrations. Only conditions where PBMC viability was significantly higher than that of both AML cell lines are shown. Bar plots show the average survival of at least three independent biological replicates (mean ± SEM). Significance of difference in survival (AML cells vs. PBMCs) was assessed via Student's *t*-test. ***: $p < 0.001$; **: $p < 0.01$; *: $p < 0.05$.

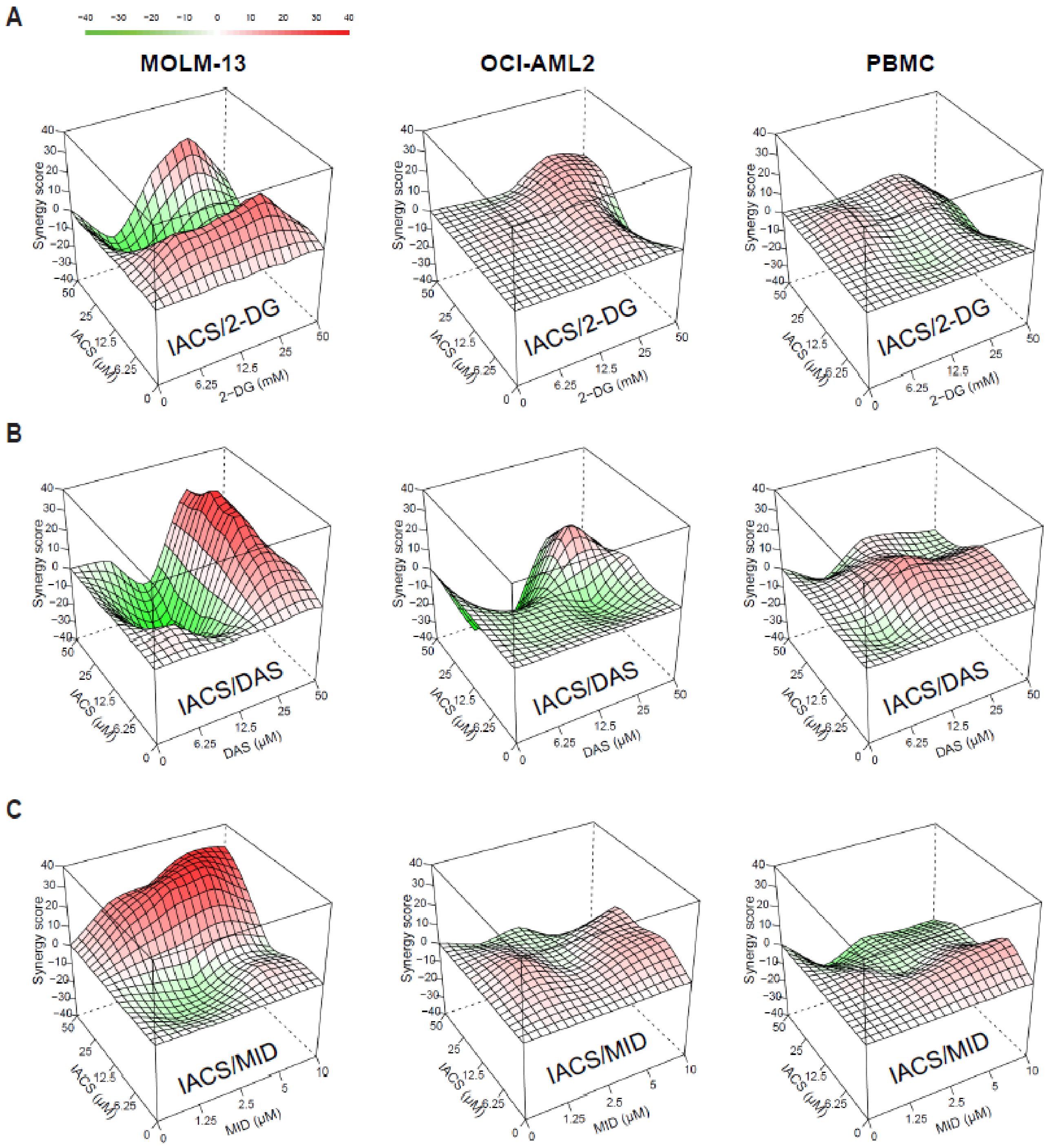

Figure S5. Synergy of selective IACS-010759-based combinations. Synergy landscapes for MOLM-13 and OCI-AML2 cell lines and healthy PBMCs after treatment. IACS-10759-based AML-selective combinations (n = 3) arranged in order of decreasing toxicity on healthy blood cells (see **Fig S4**). (**A**) IACS-010759/2-deoxy-D-glucose, (**B**) IACS-010759/dasatinib, (**C**) IACS-010759/midostaurin. Drug combination landscapes: *z*-axis, synergy score (ranges from -40 in green to +40 in red); *x*/*y*-axes, drug1/drug2 concentration range, respectively. Drug combination landscapes were built using the Bioconductor package ‘synergyfinder’. A representative replicate (with maximal synergy closest to the average value of three biological replicates) is shown.

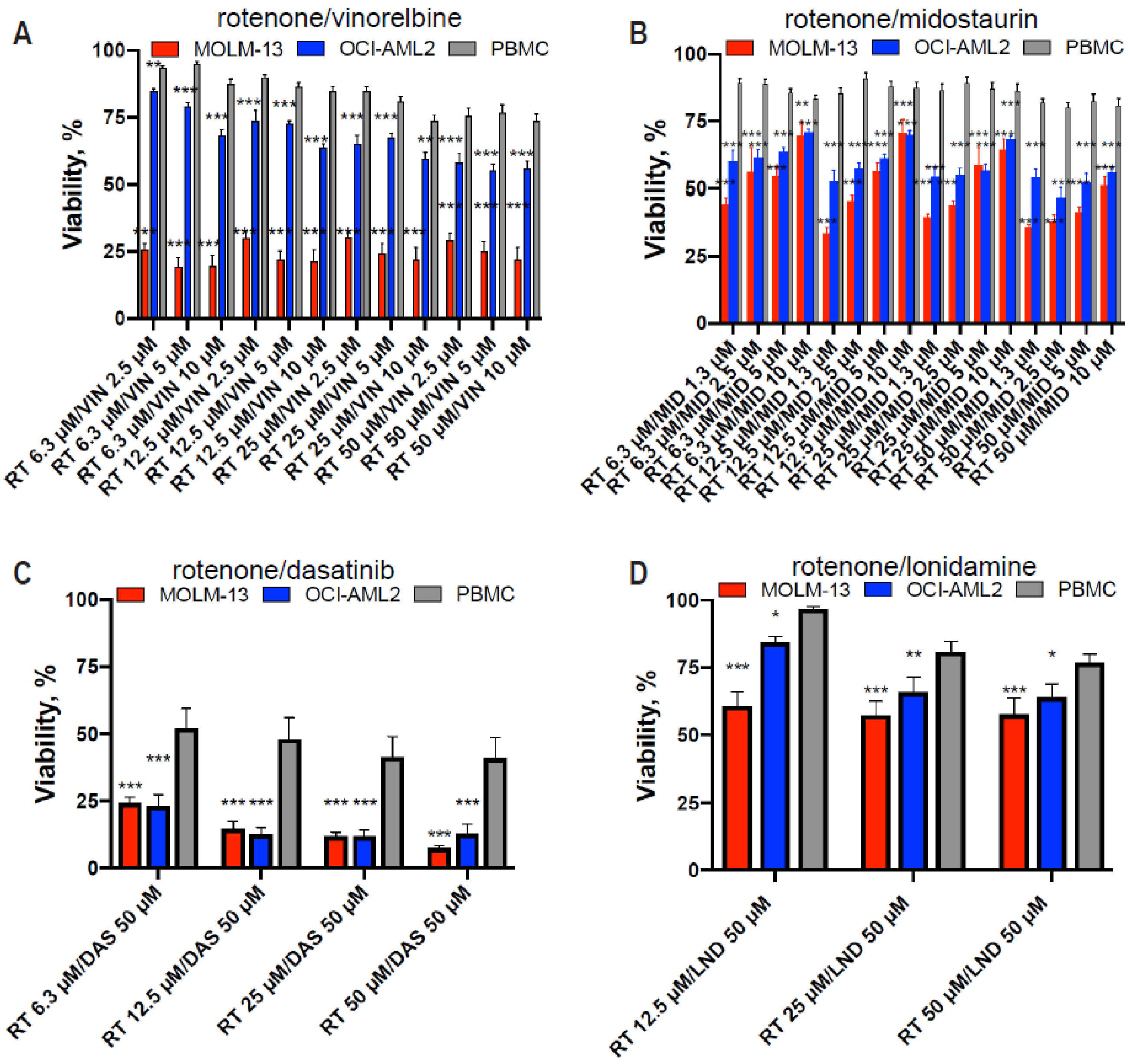

Figure S6. Rotenone-based combinations display preferential selectivity toward AML cells. Survival of MOLM-13, OCI-AML2, or healthy PBMCs following treatment with (**A**) rotenone/vinorelbine, (**B**) rotenone/midostaurin, (**C**) rotenone/dasatinib, or (**D**) rotenone/lonidamine at specified concentrations. Only conditions where PBMC viability was significantly higher than that of both AML cell lines are shown. Bar plots represent the average survival of at least three independent biological replicates (mean ± SEM). Significance of difference in survival (AML cells vs. PBMCs) was assessed via Student's *t*-test. ***: $p < 0.001$; **: $p < 0.01$; *: $p < 0.05$.

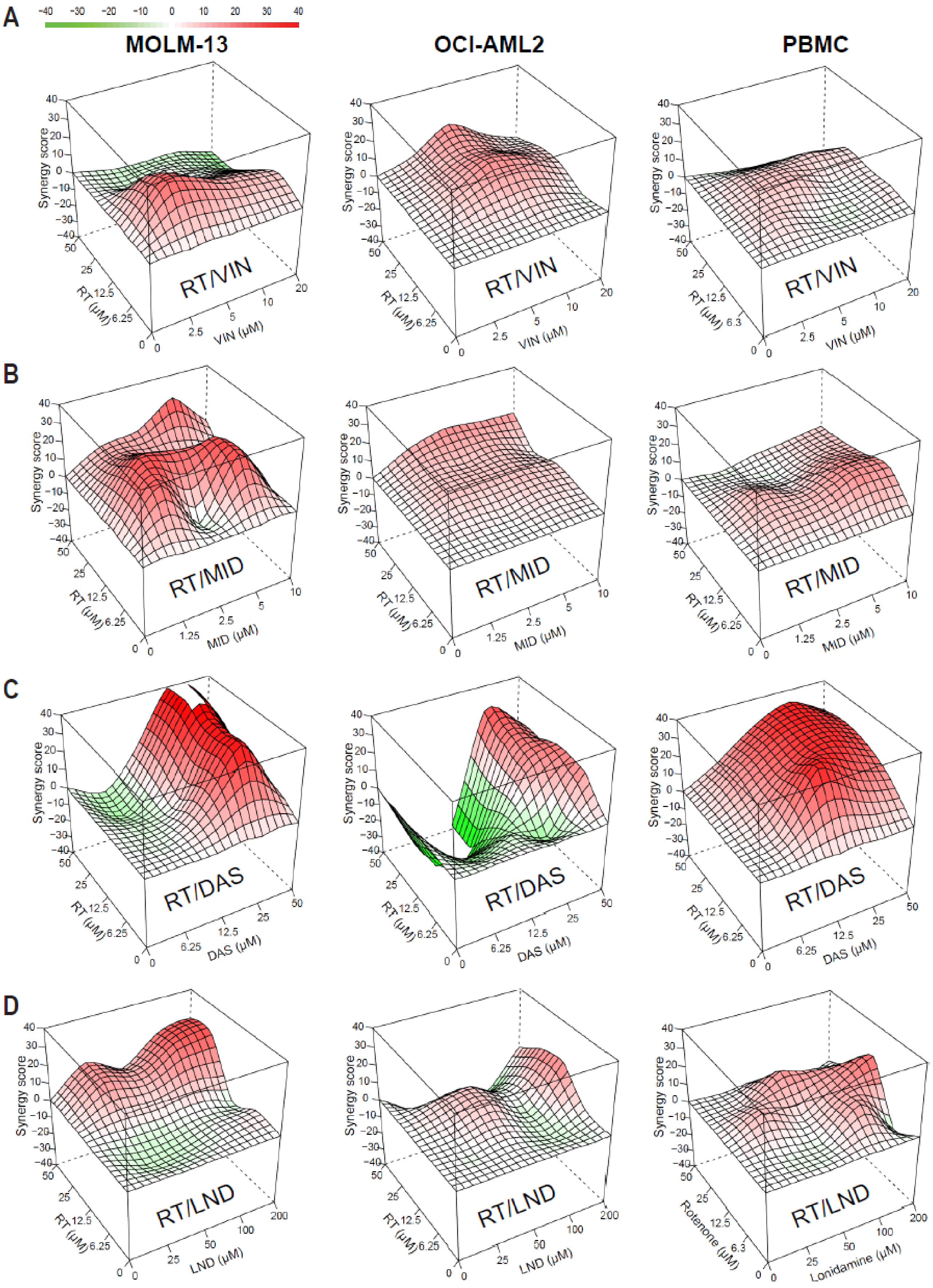
A
B
C
D
-40 -30 -20 -10 0 10 20 30 40
MOLM-13
OCI-AML2
PBMC
Synergy score
RT/VIN
RT/MID
RT/DAS
RT/LND
RT (μM)
VIN (μM)
MID (μM)
DAS (μM)
LND (μM)
Rotenone (μM)
Lonidamine (μM)

Figure S7. Synergy of selective rotenone-based combinations. Synergy landscapes for MOLM-13 and OCI-AML2 cell lines and healthy PBMCs after treatment. Rotenone-based AML-selective combinations (n = 4) arranged in same order as in **Fig S6**. (**A**) rotenone/vinorelbine, (**B**) rotenone/midostaurin, (**C**) rotenone/dasatinib, or (**D**) rotenone/lonidamine. Drug combination landscapes: *z*-axis, synergy score (ranges from -40 in green to +40 in red); *x*/*y*-axes, drug1/drug2 concentration range, respectively. Drug combination landscapes were built using the Bioconductor package 'synergyfinder'. One representative replicate (with maximal synergy closest to the average value of three biological replicates) is shown.

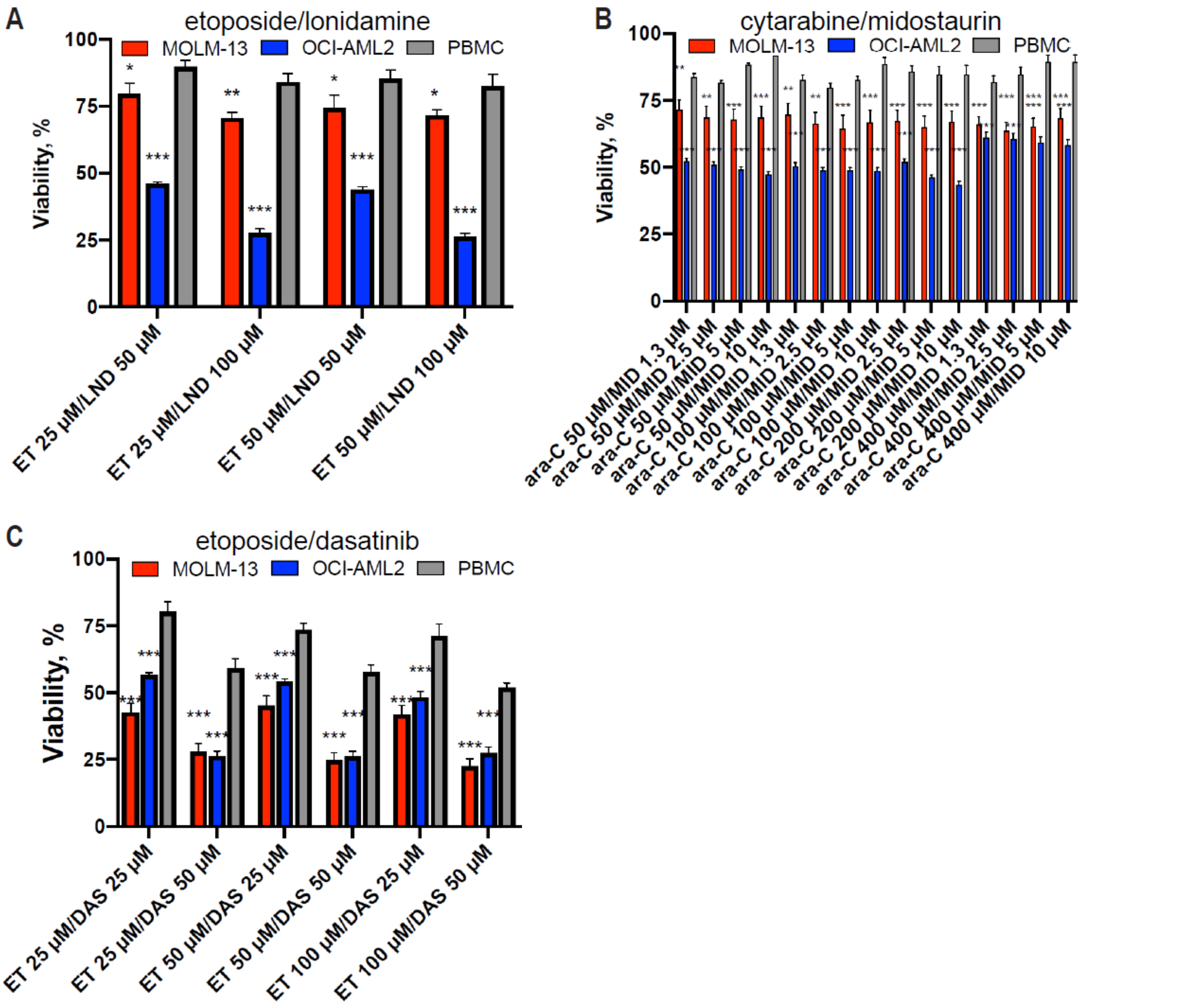

Figure S8. Etoposide- and cytarabine-based combinations display preferential selectivity toward AML cells. Survival of MOLM-13, OCI-AML2, or healthy PBMCs following treatment with (**A**) etoposide/lonidamine, (**B**) cytarabine/midostaurin, or (**C**) etoposide/dasatinib at specified concentrations. Only conditions where PBMC viability was significantly higher than that of both AML cell lines are shown. Bar plots represent the average survival of at least three independent biological replicates (mean ± SEM). Significance of difference in survival (AML cells vs. PBMCs) was assessed via Student's *t*-test. ***: $p < 0.001$; **: $p < 0.01$; *: $p < 0.05$.

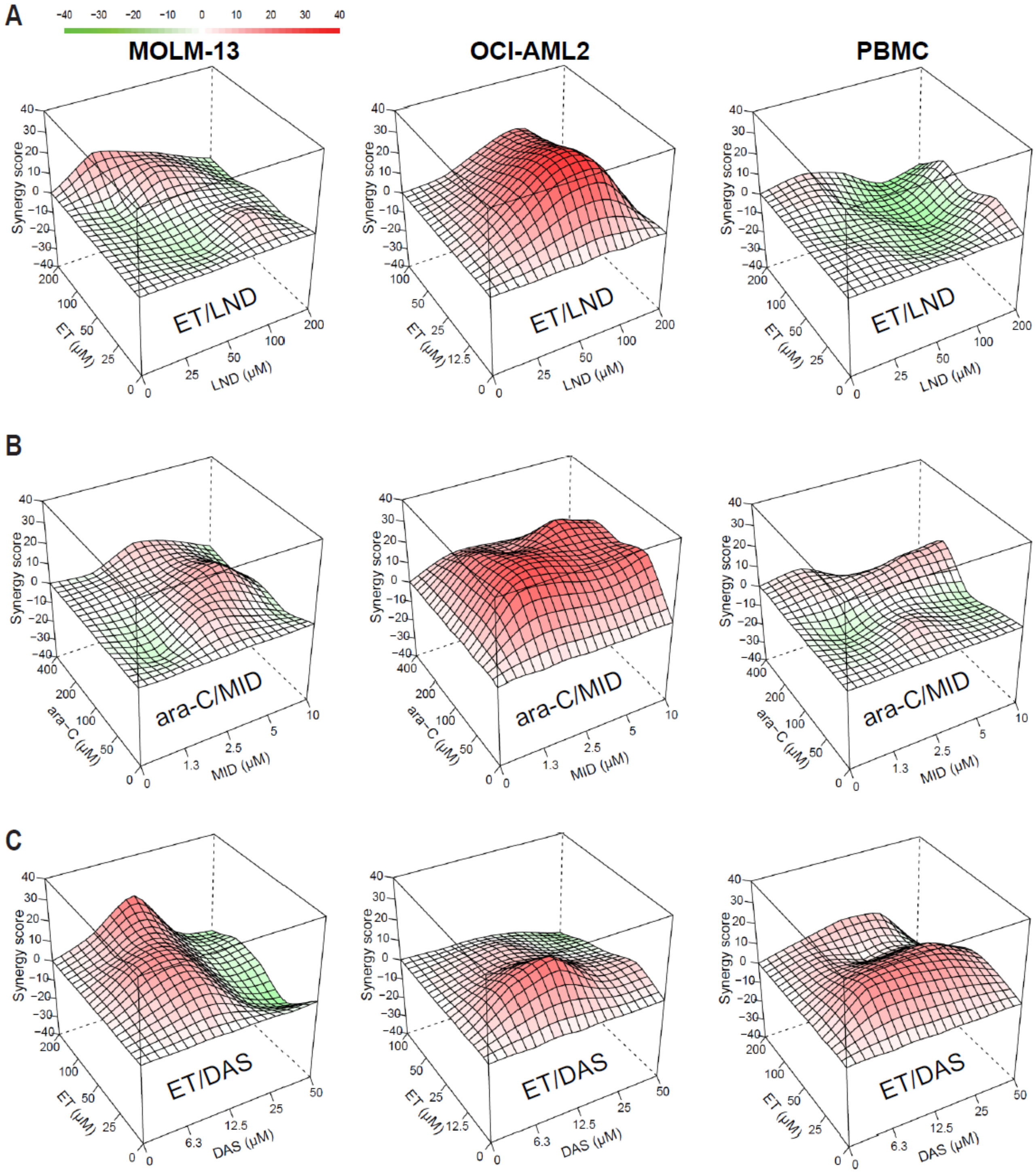

Figure S9. Synergy of selective etoposide- and cytarabine-based combinations. Synergy landscapes for MOLM-13 and OCI-AML2 cell lines and healthy PBMCs after treatment. Etoposide- and cytarabine-based AML-selective combinations (n = 3) arranged in same order as in **Fig S8**. (**A**) etoposide/lonidamine, (**B**) cytarabine/midostaurine, or (**C**) etoposide/dasatinib. Drug combination landscapes: *z*-axis, synergy score (ranges from -40 in green to +40 in red); *x*/*y*-axes, drug1/drug2 concentration range, respectively. Drug combination landscapes were built using the Bioconductor package 'synergyfinder'. One representative replicate (with maximal synergy closest to the average value of three biological replicates) is shown.

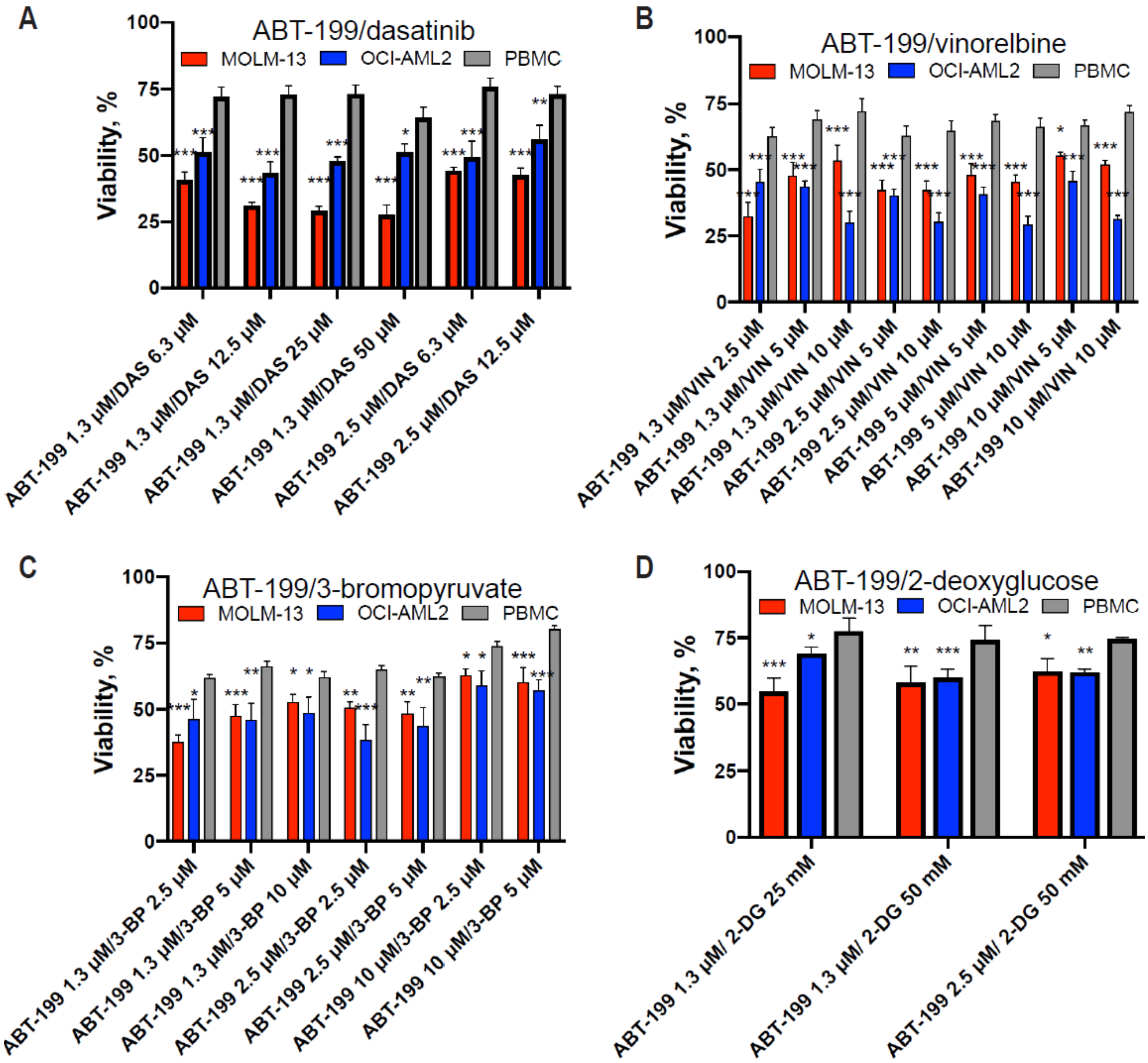

Figure S10. ABT-199-based combinations display preferential selectivity toward AML cells. Survival of MOLM-13, OCI-AML2 cells, or healthy PBMCs following treatment with (**A**) ABT-199/dasatinib, (**B**) ABT-199/vinorelbine, (**C**) ABT-199/3-bromopyruvate, or (**D**) ABT-199/2-deoxy-D-glucose at specified concentrations. Only conditions where PBMC viability was significantly higher than that of both AML cell lines are shown. Bar plots represent the average survival of at least three independent biological replicates (mean ± SEM). Significance of difference in survival (AML cells vs. PBMCs) was assessed via Student's *t*-test. ***: $p < 0.001$; **: $p < 0.01$; *: $p < 0.05$.

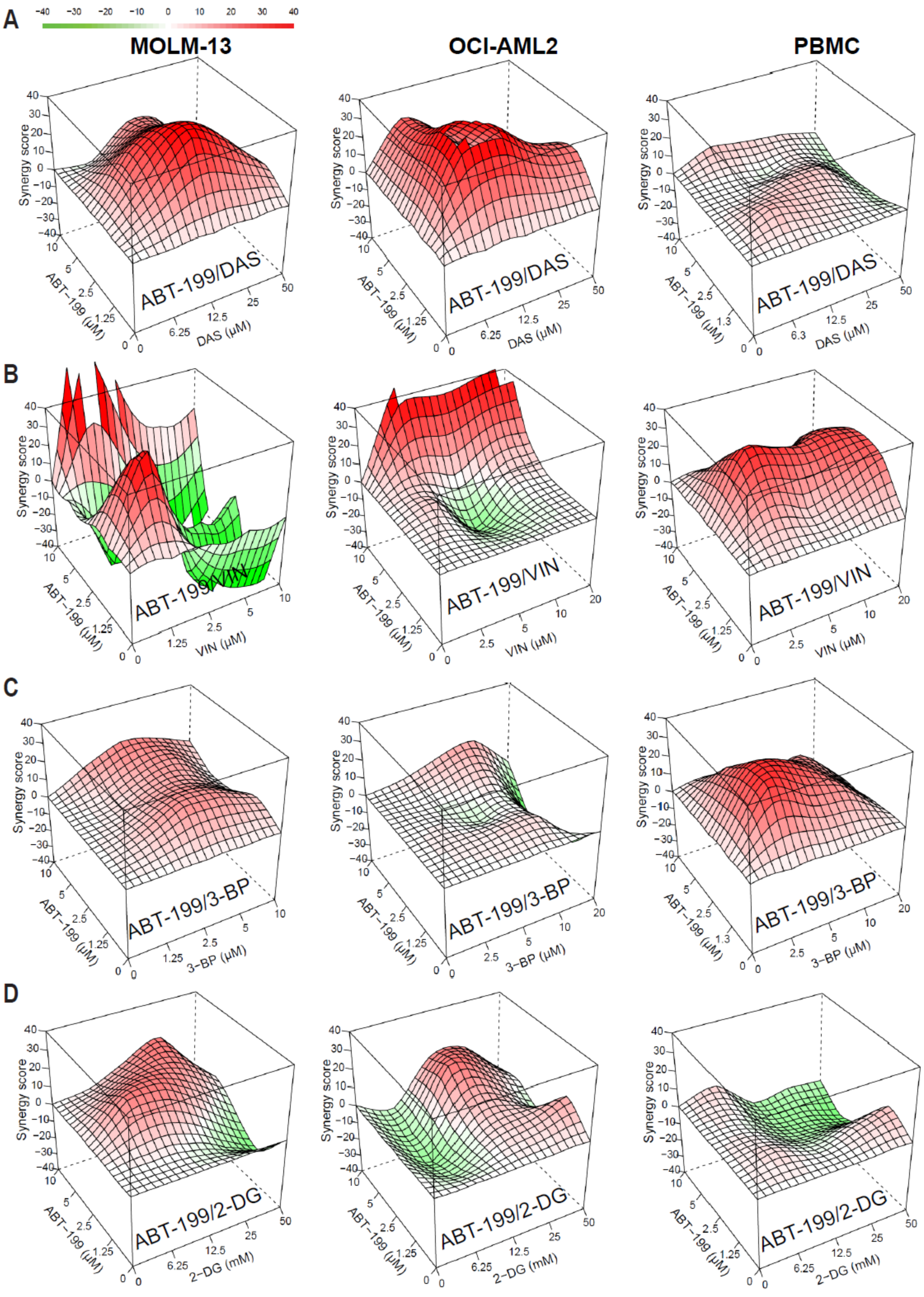
A
-40 -30 -20 -10 0 10 20 30 40
MOLM-13
OCI-AML2
PBMC
Synergy score
ABT-199/DAS
ABT-199 (µM)
DAS (µM)
B
ABT-199/VIN
VIN (µM)
C
ABT-199/3-BP
3-BP (µM)
D
ABT-199/2-DG
2-DG (mM)

Figure S11. Synergy of selective ABT-199-based combinations. Synergy landscapes for MOLM-13 and OCI-AML2 cell lines and healthy PBMCs after treatment. ABT-199-based AML-selective combinations (n = 4) arranged in same order as in **Fig S10**. (**A**) ABT-199/dasatinib, (**B**) ABT-199/vinorelbine, (**C**) ABT-199/3-bromopyruvate, or (**D**) ABT-199/2-deoxy-D-glucose. Drug combination landscapes: *z*-axis, synergy score (ranges from -40 in green to +40 in red); *x*/*y*-axes, drug1/drug2 concentration range, respectively. Drug combination landscapes were built using Bioconductor package ‘synergyfinder’. One representative replicate (with maximal synergy closest to the average value of three biological replicates) is shown.

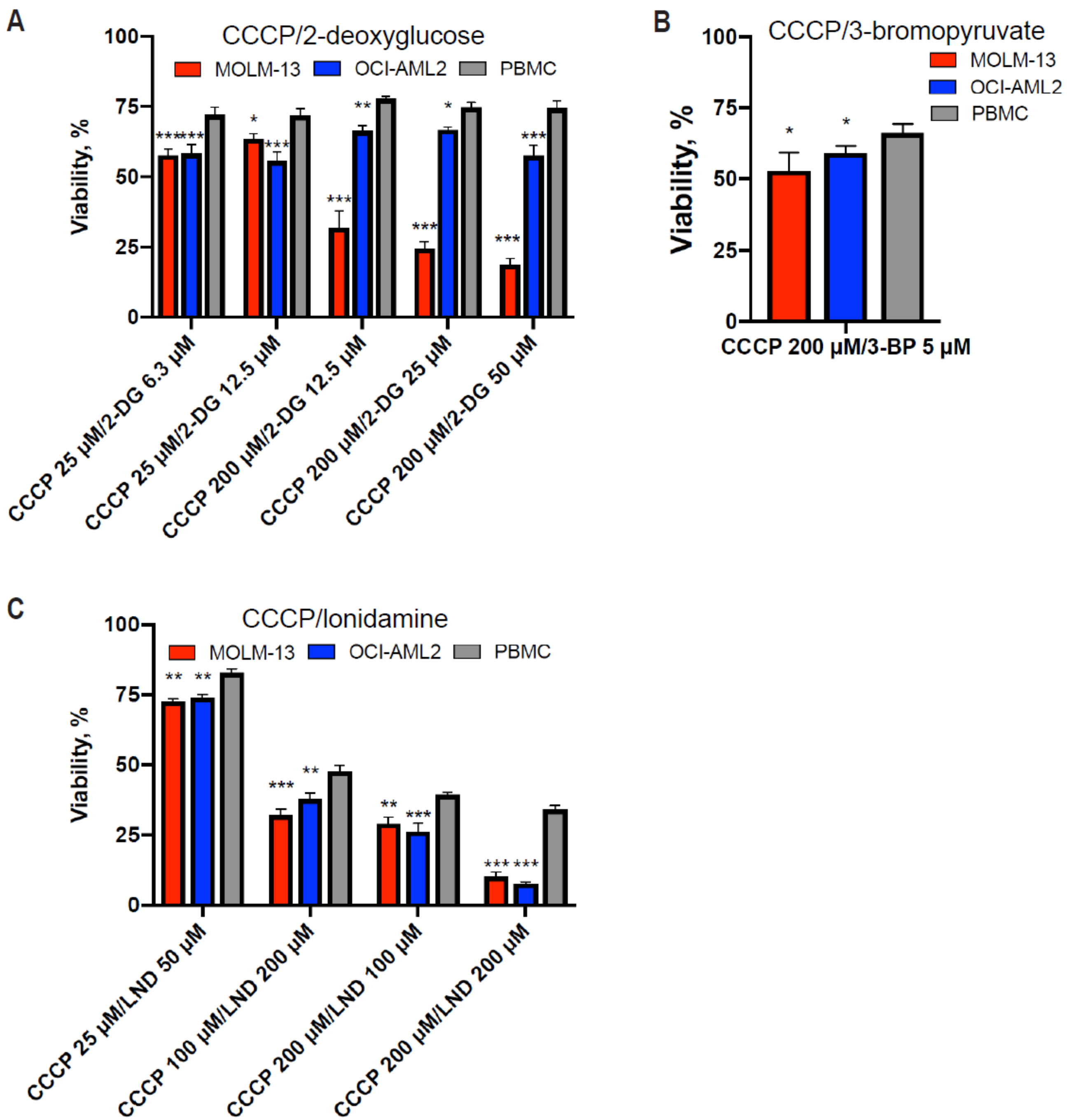

Figure S12. CCCP-based combinations display preferential selectivity toward AML cells. Survival of MOLM-13, OCI-AML2 cells, or healthy PBMCs following treatment with (**A**) CCCP/2-deoxy-D-glucose, (**B**) CCCP/3-bromopyruvate, or (**C**) CCCP/lonidamine at specified concentrations. Only conditions where PBMC viability was significantly higher than that of both AML cell lines are shown. Bar plots represent the average survival of at least three independent biological replicates (mean ± SEM). Significance of difference in survival (AML cells vs. PBMCs) was assessed via Student's *t*-test. ***: $p < 0.001$; **: $p < 0.01$; *: $p < 0.05$.

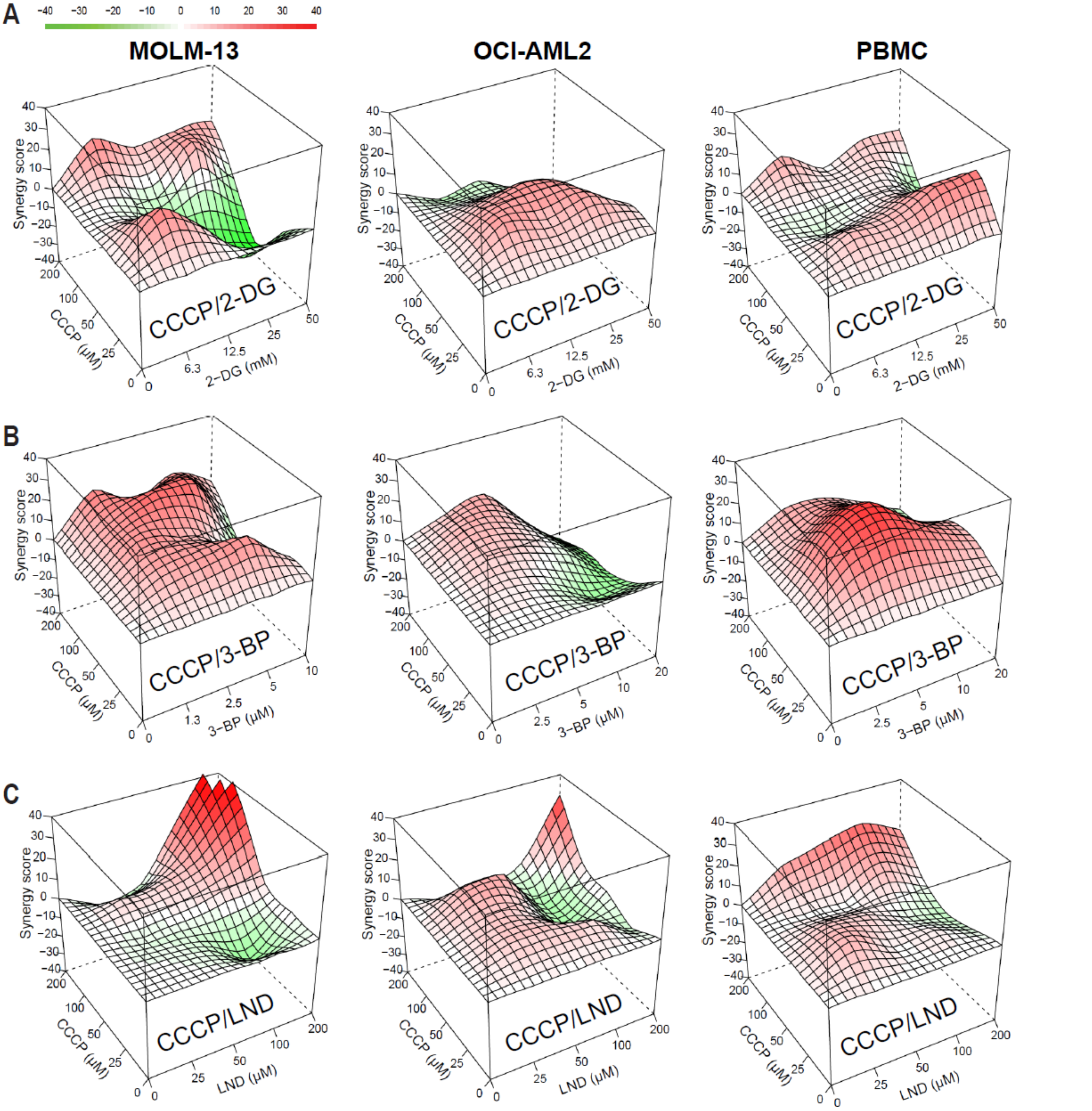

Figure S13. Synergy of selective CCCP-based combinations. Synergy landscapes for MOLM-13 and OCI-AML2 cell lines and healthy PBMCs after treatment. CCCP-based AML-selective combinations (n = 3) arranged in same order as in **Fig S12**. (**A**) CCCP/2-deoxy-D-glucose, (**B**) CCCP/3-bromopyruvate, or (**C**) CCCP/lonidamine. Drug combination landscapes: *z*-axis, synergy score (ranges from -40 in green to +40 in red); *x*/*y*-axes, drug1/drug2 concentration range, respectively. Drug combination landscapes were built using Bioconductor package 'synergyfinder'. One representative replicate (with maximal synergy closest to the average value of three biological replicates) is shown.

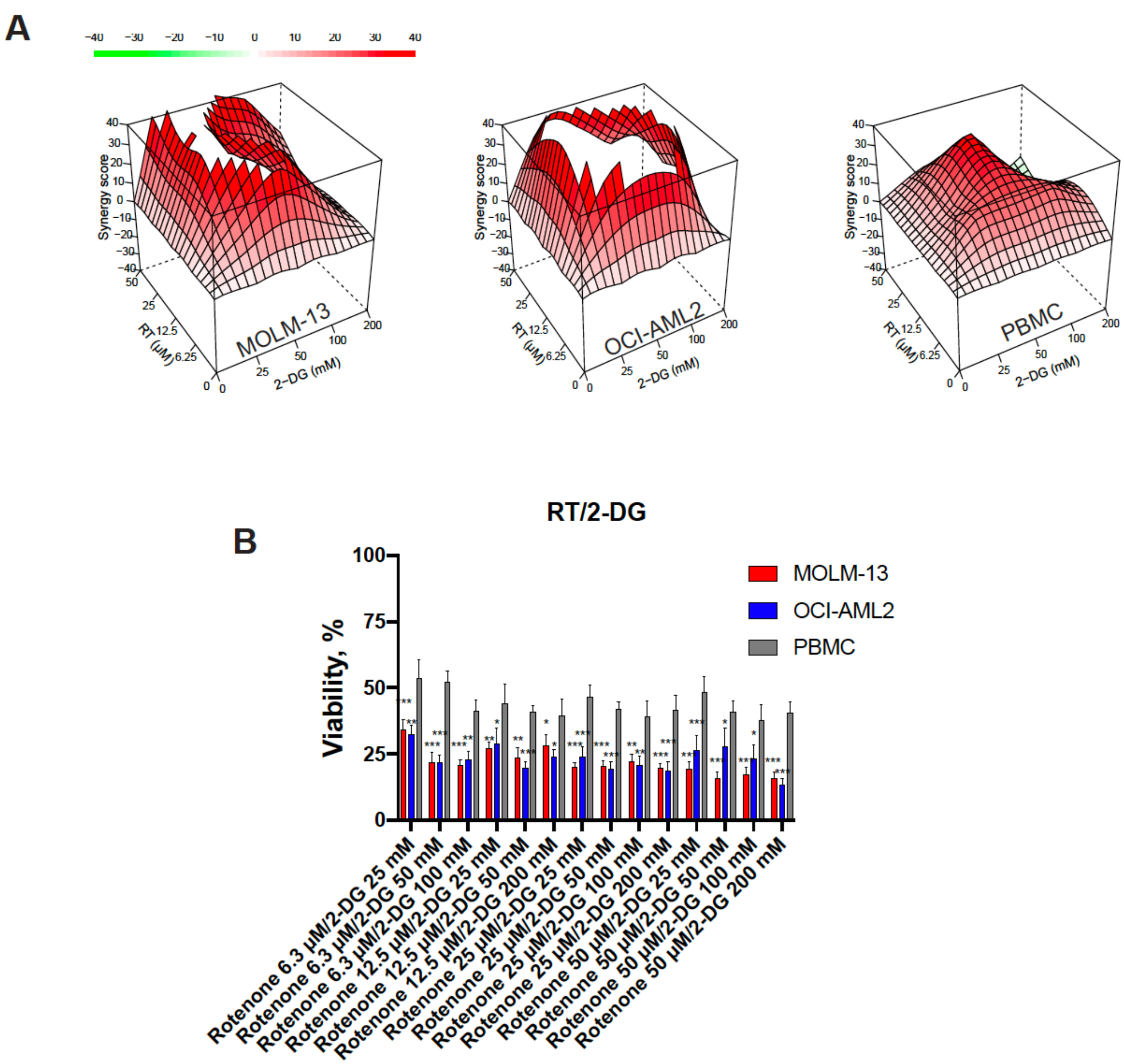

Figure S14. Trypan blue exclusion assay shows selectivity of rotenone/2-deoxy-D-glucose combination against AML cells, consistent with Hoechst/PI double-staining. (**A**) Synergy landscapes for MOLM-13 and OCI-AML2 cell lines and healthy PBMCs after treatment. Drug combination landscapes: *z*-axis, synergy score (ranges from -40 in green to +40 in red); *x*/*y*-axes, drug1/drug2 concentration range, respectively. Drug combination landscapes were built using Bioconductor package 'synergyfinder'. One representative replicate (with maximal synergy closest to the average value of three biological replicates) is shown. (**B**) Survival of MOLM-13 and OCI-AML2 cells, or healthy PBMCs following treatment with rotenone/2-DG at specified concentrations. Only conditions where PBMC viability was significantly higher than that of both AML cell lines are shown. Bar plots represent the average survival of at least three independent biological replicates (mean ± SEM). Significance of difference in survival (AML cells vs. PBMCs) was assessed via Student's *t*-test. ***: $p < 0.001$; **: $p < 0.01$; *: $p < 0.05$.

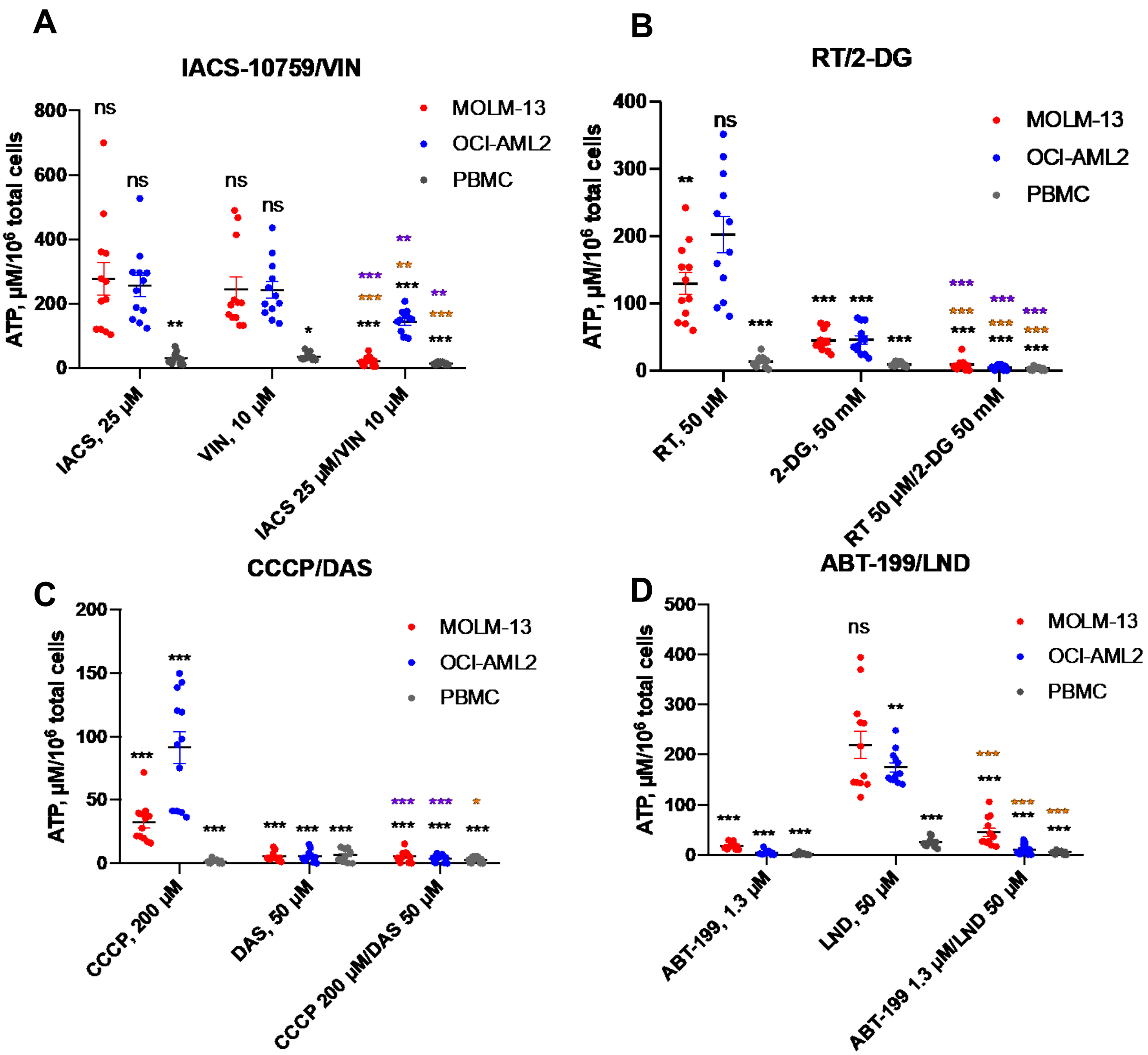

Figure S15. Changes in ATP level under single drug and combinatorial treatmens for 16 h. (**A**) IACS-010759/vinorelbine, (**B**) rotenone/2-DG, (**C**) CCCP/dasatinib, or (**D**) ABT-199/lonidamine at specified concentrations. Black stars or ns indicate comparison with untreated condition for every cell line; purple stars indicate significantly lower ATP level under combinatorial treatment compared to single mitocan for each cell line; orange stars indicate significantly lower ATP level under combinatorial treatment compared to single complementary drug for each cell line. ***: $p < 0.001$; **: $p < 0.01$; *: $p < 0.05$; ns: $p > 0.05$.

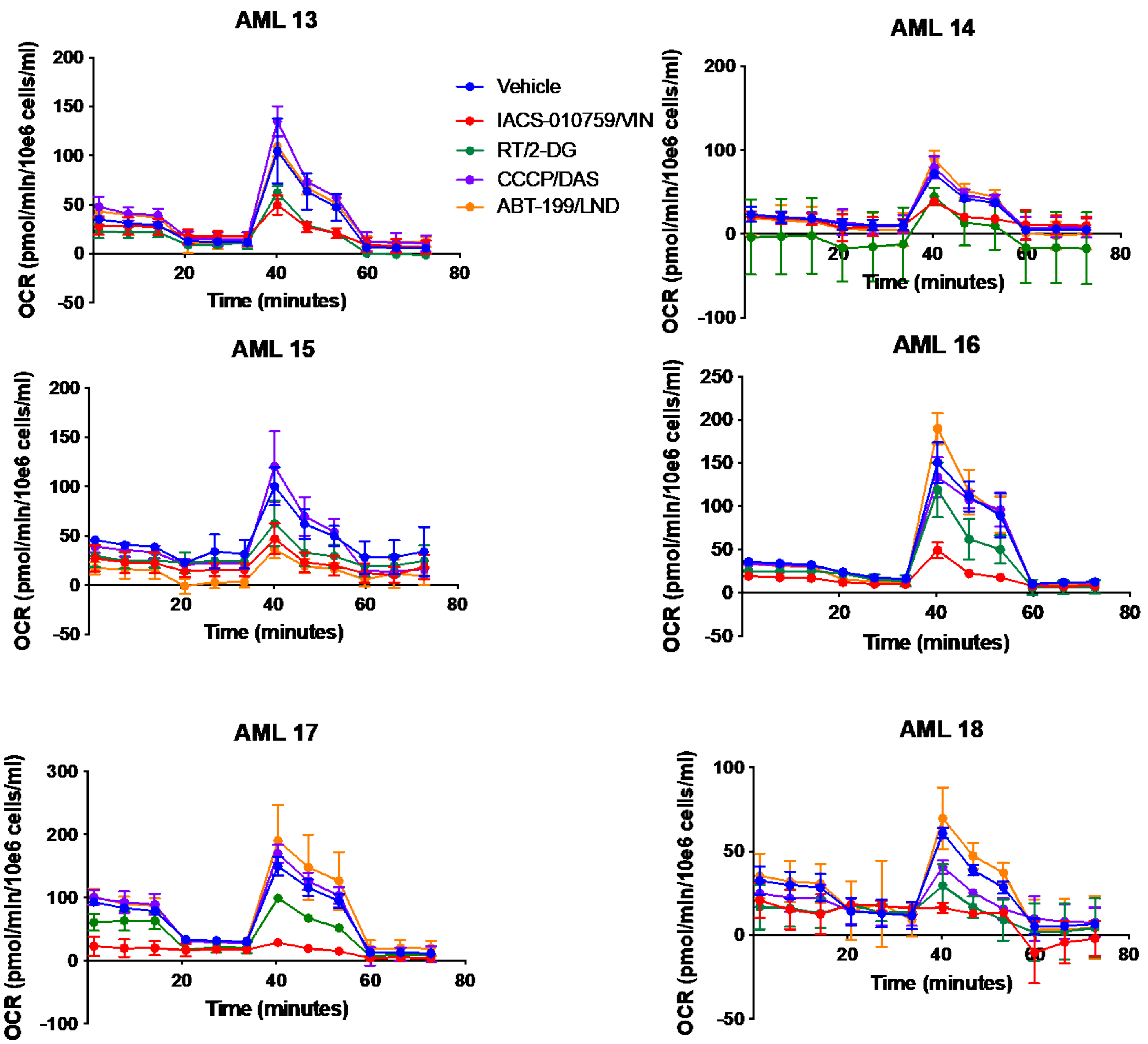

Figure S16. Bioenergetic profiling of primary AML samples (n = 6) after treatment with selected drug combinations. Oxygen consumption rate (OCR) was measured using a Seahorse flux analyzer. OCR measured in cells either untreated (blue) or treated with IACS-010759 25 nM/vinorelbine 10 nM (red), rotenone 50 nM/2-DG 50 µM (green), CCCP 200 nM/dasatinib 50 nM (purple), or ABT-199 1.3 nM/lonidamine 50 nM (orange) for 2 h. One biological replicate is shown.
